# An umbrella review of soccer small-sided games: developing an evidence-informed design model linking training components to performance outcomes

**DOI:** 10.5114/biolsport.2026.161109

**Published:** 2026-06-10

**Authors:** Süleyman Ulupınar, Serhat Özbay, Osman Ateş, Murat Turan, Kemal Coşkun, Salih Çabuk, Cebrail Gençoğlu, Adam Owen, Chris Barnes

**Affiliations:** 1 Faculty of Sports Sciences, Erzurum Technical University, Erzurum, Türkiye; 2 Faculty of Sports Sciences, Istanbul University-Cerrahpaşa, Istanbul, Türkiye; 3 Technical and Coach Education Department, Turkish Football Federation, Istanbul, Türkiye; 4 KKS Lech Poznan, Poznan, Poland; 5 Universiy of Lyon, University Claude Bernard Lyon, Villeurbanne, France; 6 CB Sports Performance Ltd, UK

**Keywords:** Soccer, SSG, Training design, Meta-Analysis, Area per player, Work-To-Rest ratio, Decision matrix

## Abstract

Small-sided games (SSGs) are widely used in soccer training to develop multiple performance domains, though interactions among design components can complicate targeting specific outcomes. This umbrella synthesis aimed to integrate findings from systematic reviews and meta-analyses to clarify how key SSG design components are associated with different training objectives and to develop an evidence-informed framework for SSG design. A synthesis of 37 systematic reviews and meta-analyses was conducted. The analysis focused on eight commonly manipulated SSG design components—Area per Player, player format, bout duration, work-to-rest ratio, rules and task constraints, touch limitation, joker usage, and directionality/goalkeeper involvement—and examined their associations with six primary performance domains: aerobic load, anaerobic/glycolytic demands, high-speed running and sprint exposure, neuromuscular/mechanical demands, technical execution, and tactical behaviors. Consistent patterns indicate that spatial density and player format are central determinants of SSG responses. Aerobic strain is most frequently reported in formats ranging from 2 v 2 to 4 v 4, with approximately 100–150 m^2^ per player. In contrast, maximal sprint exposure is rarely achieved unless pitch dimensions exceed ~300–365 m^2^ per player, suggesting the need for complementary conditioning when maximal speed development is required. Smaller formats and restricted spaces are associated with higher acceleration–deceleration density and increased technical involvement, while rule manipulations can substantially increase internal load and game intensity. Tactical behaviors were particularly sensitive to numerical balance, directional constraints, and the use of jokers. The synthesized evidence was translated into an evidence-informed SSG design matrix that links key training components to targeted performance outcomes.

## INTRODUCTION

Small-sided games (SSGs) are widely used in soccer training because they simultaneously stimulate physiological, physical, technical, and tactical adaptations in representative game environments [1–3]. However, the complex interactions among multiple design constraints often make it difficult for practitioners to intentionally target specific training outcomes. Within a constraints-led approach, SSG design variables can be conceptually organized into four broad categories: spatial constraints (e.g., area per player and pitch dimensions), temporal constraints (e.g., bout duration and work-to-rest ratio), rule-based constraints (e.g., touch limitations, scoring rules, and directional play), and informational constraints (e.g., joker usage or numerical imbalances that alter decision-making contexts) [4–7].

Although previous studies have documented the effects of individual SSG variables on selected physiological or performance outcomes, the evidence remains fragmented across multiple reviews [1, 3, 8, 9]. Importantly, these relationships are not always linear. For example, changes in format size or spatial density may produce different physiological or mechanical responses depending on the interaction between player numbers and area per player [10–13]. Consequently, recommendations derived from the literature should be interpreted conditionally within the context of the interaction between format and spatial density, rather than as universal prescriptions.

From a practical training perspective, SSG manipulation typically relies on a limited number of high-impact design levers. Drawing on principles from constraints theory, spatial and temporal constraints (e.g., area per player, pitch dimensions, and bout duration) can be considered primary regulators of physical and physiological load, whereas rule-based and informational constraints (e.g., touch limitations, joker usage, and scoring rules) function as secondary modifiers that shape technical and tactical behaviors [1, 14–16]. Recognizing this hierarchy may help practitioners prioritize which variables to adjust first when targeting specific performance outcomes.

Because SSG manipulation often involves trade-offs between competing objectives, it is also important to acknowledge that a single design change may enhance one performance aim while simultaneously reducing another [16, 17]. For instance, smaller formats may increase acceleration–deceleration density and technical involvement but reduce opportunities for high-speed running [6, 18–20]. To facilitate interpretation of these interactions, the synthesis framework distinguishes between different layers of performance capacity. In the present study, performance capacities are operationalized across six domains: aerobic/internal load (commonly assessed via heart rate responses), anaerobic and glycolytic demands (e.g., blood lactate or repeated sprint activity), high-speed running and sprint exposure (distance or time above velocity thresholds), acceleration–deceleration density and change-of-direction actions, technical execution (e.g., passes, dribbles, and shots), and tactical behaviors (e.g., team organization, spacing, and collective decision-making).

The present study aims to synthesize available evidence from systematic reviews and meta-analyses to clarify how key SSG design components are associated with different performance objectives in soccer. Using an umbrella synthesis approach, findings from existing evidence syntheses were integrated to identify consistent relationships between commonly manipulated SSG variables and their reported training responses. Particular attention was given to moderators that repeatedly emerge in the literature as major determinants of SSG outcomes, including area per player, player format, bout duration, and rule constraints. These variables are consistently reported as central regulators of physiological load, movement demands, and technical–tactical behaviors during SSGs. By organizing this evidence into a structured framework, the study aims to translate accumulated research findings into an evidence-informed model linking SSG training components with targeted performance outcomes.

## MATERIALS AND METHODS

### Literature Search Strategy

A comprehensive literature search was conducted to identify systematic reviews and meta-analyses examining the physiological, physical, and performance-related responses associated with SSGs and related game-based training formats in soccer (Figure 1). The search strategy was developed according to established evidence-synthesis recommendations for umbrella reviews and aimed to capture the wide range of terminology used to describe small-sided and modified game formats in soccer training.

**FIG. 1:**
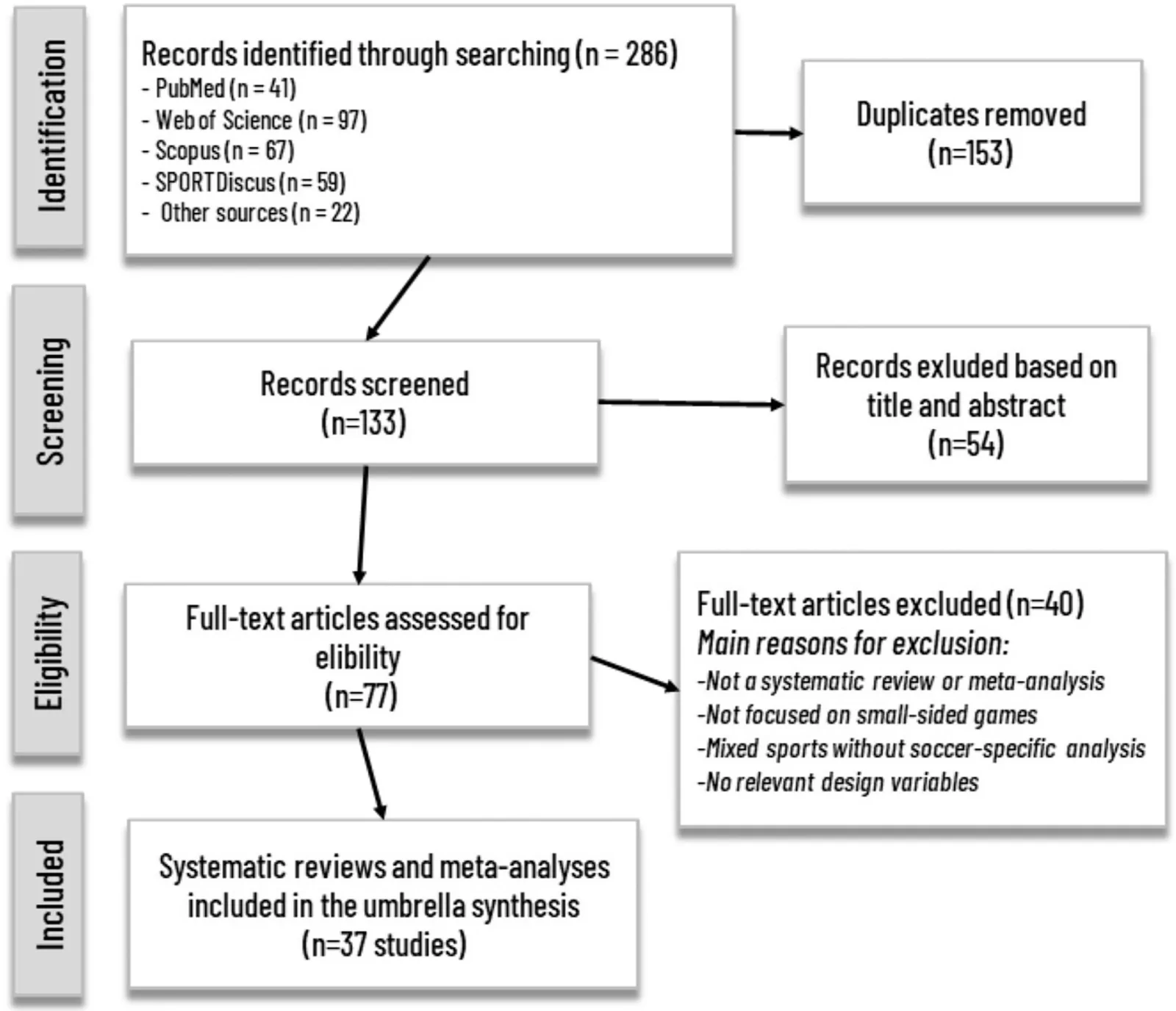
PRISMA flow diagram.

Electronic searches were performed in four databases: PubMed, Web of Science, Scopus, and SPORTDiscus. In addition, complementary searches were conducted through manual screening of reference lists from relevant articles and additional targeted searches to identify potentially eligible studies not captured during the database searches.

The search strategy incorporated multiple synonymous descriptors for small-sided games (e.g., small-sided games, modified games, conditioned games, and related training formats), combined with terms related to soccer and evidence synthesis (e.g., systematic reviews and meta-analyses). The complete database-specific Boolean search strategies are provided in Supplementary Material S1.

The search process identified 286 records across all sources. After removing 153 duplicate records, 133 studies remained for title and abstract screening. During this stage, 54 records were excluded based on title and abstract evaluation (Supplementary Material S2).

Subsequently, 77 full-text articles were assessed for eligibility. Forty studies were excluded at this stage because they were not systematic reviews or meta-analyses, did not specifically focus on soccer small-sided games, involved mixed team sports without soccerspecific analysis, or did not examine relevant SSG design variables.

Following the eligibility assessment, 37 systematic reviews and meta-analyses met the inclusion criteria and were incorporated into the umbrella synthesis [1–10, 12, 13, 15–39].

These studies collectively examined a wide range of outcomes related to internal load (e.g., heart rate and perceived exertion), external load indicators (e.g., total distance, high-speed running, sprinting, and acceleration–deceleration profiles), physical performance adaptations (e.g., ${\dot{\text{V}}\text{O}}_{2\max}$, sprint performance, and repeated-sprint ability), and contextual training modifiers such as pitch dimensions, area per player, player numbers, bout duration, and rule constraints. This body of evidence formed the empirical basis for the development of the evidence-informed SSG design framework proposed in the present study.

### Eligibility Criteria (PICOS Framework)

The eligibility criteria for the umbrella synthesis were defined according to the Population, Intervention, Comparison, Outcomes, and Study Design (PICOS) framework. The population included soccer players of all sexes, ages, and competitive levels. Reviews that included multiple team sports or mixed sport samples were also considered eligible when they contained information relevant to the aims of the present study, such as analyses of small-sided game formats, pitch dimensions, or design variables applicable to soccer training contexts. In such cases, studies were included when the findings provided conceptual or practical insights relevant to soccer SSG design, even if the original review examined more than one sport.

The intervention of interest consisted of SSGs and modified gamebased training formats used in soccer training. These included variations in player numbers, pitch dimensions, relative player area, bout duration, work-to-rest ratio, and rule manipulations commonly used to modify SSG training stimuli.

Because the purpose of the present study was to synthesize design principles rather than to evaluate a specific intervention effect, a direct-comparison condition was not required. Reviews comparing SSGs with other training methods (e.g., high-intensity interval training or running-based conditioning) were eligible, as were reviews examining the effects of manipulating SSG design variables within SSG formats.

The outcomes of interest included physiological responses (e.g., heart rate or internal load), external load indicators (e.g., total distance, high-speed running, sprint exposure, and acceleration–deceleration profiles), physical performance adaptations (e.g., aerobic capacity or repeated-sprint ability), as well as technical and tactical performance indicators.

Finally, regarding study design, only systematic reviews and meta-analyses examining SSG-related training responses were included in the umbrella synthesis. Narrative reviews, methodological papers, conference abstracts, and primary experimental studies were excluded.

### Data Extraction and Evidence Synthesis

Data extraction was conducted exclusively at the level of systematic reviews and meta-analyses, consistent with the umbrella synthesis design of the present study. Individual experimental studies were not independently analyzed; instead, evidence was synthesized from higher-level sources to identify recurring relationships between SSG design variables and performance-related outcomes. The extraction process followed a structured evidence-mapping framework summarized in Table 1, which presents the characteristics and key findings of the included reviews.

**TABLE 1:** Characteristics and Key Findings of Systematic Reviews and Meta-Analyses Included in the Umbrella Synthesis.

Study (Author, Year)	Type of Review	Population	SSG Characteristics	Main Outcomes / Key Findings	Implications for SSG Design
Clemente & Sarmento (2021) [15]	Systematic Review	105 male soccer players (youth, semi-professional, and professional; ages 13.9–25.6) from 5 studies.	Formats: 3 v 3 to 8 v 8; Pitches: 25 × 20 m to 64 × 40 m; Combined with running-based HIIT or drills.	Acute: Combined training (SSG+running) significantly increased RPE, blood lactate, and high-intensity running distance (> 14.4 km/h and > 19.8 km/h) compared to SSG-only. Adaptations: Both combined and SSG-only methods showed similar improvements in aerobic performance (e.g., ${\dot{\text{V}}\text{O}}_{2\mathrm{peak}}$ increased by 5.5% in the combined vs 1.6% in the SSG-only in one study). No significant benefits were found for sprinting, jumping (CMJ), or COD.	SSGs often limit players’ exposure to high-intensity running (> 19.8 km/h) due to limited longitudinal pitch space. Practitioners should supplement SSGs with running-based HIIT to ensure mechanical loading and high-speed stimuli. The order of combination (SSG first or HIIT first) does not affect adaptations, but higher session frequency significantly enhances aerobic outcomes.

Clemente et al. (2020) [22]	Systematic Review	Male soccer players across various age groups (U-9 to Seniors) and expertise levels (amateur to professional) from 34 reviewed studies.	Variables: Formats (1 v 1 to 10 v 10), pitch dimensions (relative area and L:W ratio), task constraints (floaters, scoring methods, GKs), and multidimensional factors (fatigue, instructions).	Format Effects: Smaller formats (e.g., 3 v 3) significantly increase individual tactical behaviors, such as penetration and defensive delays. Larger formats (e.g., 5 v 5+) enhance collective behaviors such as attacking/defensive unity and positional regularity. Pitch Constraints: Larger pitches increase spatial exploration variability and passing sequence length. Player Factors: Older and elite players occupy pitch width more effectively and show greater sensitivity to pitch-size changes in their positioning.	SSG design must align with specific tactical outcomes: use small formats for individual duels and large formats for team coordination. Coaches should use offensive/defensive instructions to effectively modulate player dispersion and compactness. Tactical complexity should be adjusted for age and skill level, as developmental players may focus more on longitudinal exploration, while elite players use the wings more effectively.

Clemente et al. (2021) [1]	Systematic Review and Meta-analysis	172 young team sports players (under 18 years old) from 6 studies (mostly soccer, one volleyball).	Formats: 1 v 1 to 3 v 3 (with some up to 4 v 2); Pitch sizes: 10 × 20 m to 20 × 30 m (soccer) and 7 × 3 m to 12 × 6 m (volleyball); Individual area per player: 5–100 m^2^; Duration: 5–12 weeks.	Technical Execution: Small significant effect favoring SSGs over control groups (ES = 0.59; p < 0.001). Within-group analysis showed moderate improvements for SSG (ES = 1.06; p < 0.001) vs. controls (ES = 0.65; p = 0.001). Tactical Behavior: No eligible studies reported tactical behavior outcomes.	Small formats (1 v 1 to 3 v 3) are highly effective for increasing ball engagement and providing variable stimuli for technical development. Practitioners should prioritize SSGs over generic training to boost technical skill acquisition. However, traditional closed-skill agility tests may not fully capture the tactical-technical integration of SSGs; therefore, the use of valid in-game-based assessment instruments is recommended.

Clemente et al. (2021) [17]	Umbrella Review of SRs and SRMAs	Youth and adult athletes across team ball sports (predominantly soccer, but also basketball and rugby).	Variables: Format (1 v 1 to 10 v 10), pitch configuration (ApP, length-to-width ratio), scoring methods (GK involvement, targets), touch limits, and tactical instructions (e.g., man-to-man marking).	Aerobic Capacity: Consistently positive effects on ${\dot{\text{V}}\text{O}}_{2\max}$ (ES = 1.94). Neuromuscular Performance: Inconsistent findings for jumping, linear sprinting, and COD (ES ranging from -0.89 to 1.19). Constraints: Smaller formats and larger pitches consistently increase internal load; intermittent regimens increase external load.	SSGs are powerful pedagogical tools for improving aerobic fitness and tactical/technical behaviors concurrently. However, due to heterogeneous neuromuscular adaptations, SSGs should be supplemented with complementary training methods (e.g., HIIT or strength training) to optimize speed and power. Practitioners should favor intermittent regimens to intensify external load.

Clemente et al. (2021) [24]	Systematic Review and Meta-analysis	156 male youth soccer players (12 groups from 6 parallel studies).	Formats: 2 v 2 to 4 v 4; Pitch length: 18–40 m; Individual area per player: 50–150 m^2^; Bout duration: 2.5–4 min.	Sprinting: HIIT was significantly better than SSGs (Between-group ES = 0.42, p = 0.012). COD: No significant difference between groups (ES = 0.85, p = 0.175), but HIIT showed significant within-group improvement (ES = 1.04, p = 0.005) while SSGs did not. Jumping (VHJ): No significant difference between HIIT and SSGs (ES = 0.25, p = 0.474).	SSGs underexpose players to high-speed running and sprinting because of the limited longitudinal pitch distance. While effective for aerobic goals, SSGs alone are insufficient for improving linear speed and explosive power. Practitioners should supplement SSGs with running-based HIIT and strength/power training (e.g., plyometrics) to address these specific neuromuscular demands.

Clemente et al. (2021) [25]	Systematic Review and Meta-analysis	77 male soccer players (youth and university-level; mean age 14.2–19.0).	Formats: 2 v 2 to 4 v 4; Pitch: 20 × 15 m to 40 × 50 m; Area per player (ApP): 75–100 m^2^; Work duration: 45 s to 4 min	RSA: No significant difference between SSG and HIIT (ES = 0.30; p = 0.181). Within-group analysis showed neither SSG (ES = -0.23; p = 0.697) nor HIIT (ES = 0.08; p = 0.899) significantly improved RSA.	SSGs alone, especially in smaller formats/pitches (ApP 75–100 m2), may restrict sprinting demands. Complementary training (e.g., strength/power or combined HIIT) is required to effectively improve RSA.

Clemente et al. (2021) [26]	Systematic Review and Meta-analysis	Healthy youth and young adult male soccer players (pooled n=75 for physical; n=46 for tactical analysis).	Formats: 3 v 3 to 6 v 6; Pitch sizes: 30 × 20 m to 62 × 43 m; Mental fatigue induced via 20–30 min protocols (primarily Stroop task) immediately prior to SSGs.	Physical: No significant effect of mental fatigue on total running distance (ES = 0.13; p = 0.307). Tactical: No significant overall effect on tactical behavior (ES = 0.56; p = 0.079), though high heterogeneity (I^2^ = 85.5%) was noted, with some studies showing reduced accuracy in offensive unity (-26.5%).	Coaches can implement cognitive-heavy tasks (e.g., video analysis sessions) before training without acutely impairing running volume or general tactical execution in SSGs. However, because mental fatigue may increase perceived exertion, practitioners should exercise caution during congested schedules to avoid compromising long-term recovery.

Clemente et al. (2022) [21]	Systematic Review	24 studies involving male soccer players across various levels (amateur, professional, and youth aged U12 to Seniors).	Formats: 1 v 1 to 7 v 7. Variables: Intra- and inter-session variability of internal load (HR, RPE, Lactate), external load (TD, HSR, ACC, DEC, Player Load), technical actions, and tactical behaviors.	Low Variability: Internal load (e.g., %HRmax CV: ~0.5–6%) and low-speed external load (e.g., TD CV: ~5–9%) are highly stable. High Variability: High-speed external loads (CV: ~45–146%) and technical actions (CV often > 40%) show substantial variation. Moderators: Lactate and RPE are more variable than heart rate.	SSGs are reliable tools for cardiorespiratory (aerobic) conditioning because of their stable heart rate responses. However, they provide inconsistent stimulus for high-speed running; therefore, high-intensity running should be supplemented with analytical running drills to ensure standardized mechanical overload. Coaches should view high technical/tactical variability as a natural feature that fosters creativity rather than a negative training aspect.

Clemente et al. (2023) [8]	Systematic Review and Meta-analysis	Soccer players of any age, sex, or skill level (pooled n up to 1,035 across 41 studies).	Formats: 1 v 1 to 11 v 11; Relative area per player: 15.6–199.75 m^2^ (smaller) vs. 37.5–334 m^2^ (larger).	Physiological/Physical: Larger pitches significantly induced greater HR (ES=0.50, p < 0.001), RPE (ES=0.70, p < 0.001), Total Distance (ES=1.95, p < 0.001), and HSR (ES=1.20, p < 0.001). Tactical: Larger pitches significantly increased Stretch Index (ES=1.02, p < 0.001) and Surface Area (ES=1.54, p < 0.001). No significant differences for ACC, DEC, passes, or dribbles.	Larger pitch sizes (> 250 m^2^/player) are recommended for mid-week sessions (MD-3) to maximize physiological and physical loading (TD and HSR). Smaller pitches should be used for tactical exploration in youth or low-skill groups as they reduce task complexity and are ideal for tapering or compensatory sessions (MD-2 or MD+1) due to lower mechanical loading.

Clemente et al. (2024) [23]	Systematic Review and Meta-analysis	Male and female youth and adult soccer players (minimum PCF Tier 2) from 20 studies; median n=21 per study.	Formats: 1 v 1 to 10 v 10; Relative area per player: ≤ 100 m^2^ (10 studies) vs. > 100 m^2^ (4 studies); Bout duration: ≤ 4 min (13 studies).	Endurance: Non-significant small effect favoring HIIT (ES = 0.37, p = 0.074). ${\dot{\text{V}}\text{O}}_{2\max}$: Non-significant small effect favoring SSGs (ES = -0.20, p = 0.303). Within-group: Both SSG and HIIT significantly improved endurance (SSG ES = 0.70; HIIT ES = 0.94) and ${\dot{\text{V}}\text{O}}_{2\max}$ (SSG ES = 0.50; HIIT ES = 0.34–0.45).	SSGs and HIIT provide comparable chronic adaptations for endurance and ${\dot{\text{V}}\text{O}}_{2\max}$. While small formats (1 v 1–4 v 4) effectively challenge maximal aerobic power, they may not provide sufficient running load. To optimize endurance design, practitioners should consider larger formats or combine SSGs with intermittent runs (e.g., 15s:15s) to ensure adequate locomotor overload while maintaining tactical specificity.

Clemente et al. (2025) [18]	Systematic Review and Meta-analysis	1,767 soccer players (male and female; youth and adult; Tier 2 to Tier 5) from 72 studies.	Formats: 1 v 1 to 10 v 10; Median relative area: 100 m^2^/player; Includes variations with goalkeepers, small goals, and ball possession.	Physiological: Formats < 4 v 4 (especially 2 v 2 and 3 v 3) are significantly more intense. RPE was higher in smaller formats (Hedges’ g: -1.917 to -0.524; p < 0.01). Blood lactate (BLa) was significantly higher in 2 v 2 and 3 v 3 vs. larger formats. Physical: 4 v 4 and 3 v 3 covered significantly more total distance (TD) than 2 v 2 (ES=2.6–4.0; p < 0.01). 4 v 4 induced the highest distance at speeds > 18 km/h. No significant differences in ACC/DEC across formats. Technical: 2 v 2 and 3 v 3 promoted better ball possession than 4 v 4. 3 v 3 elicited the highest frequency of successful shots and dribbles compared to 4 v 4, 5 v 5, and 6 v 6.	SSG formats smaller than 4 v 4 (specifically 2 v 2 and 3 v 3) should be used to target aerobic power and anaerobic contribution, given their higher metabolic strain. Mid-sized formats (4 v 4 to 6 v 6) are most effective at meeting high locomotor demands (total distance and high-speed running) because they provide sufficient longitudinal space. The 3 v 3 format is uniquely effective for technical development, offering the best balance for ball possession, dribbling, and shooting success.

Coito et al. (2020) [12]	Systematic Review	1,035 soccer players from 31 studies (published 2011–2019).	Formats: 1 v 1 to 10 v 10; varied pitch sizes (e.g., 20 × 16 m to 91 × 63 m); constraints: numerical relations, scoring targets, and touch limits.	Variables: Identified 21 positional variables grouped into team balance (e.g., Centroid), playing space (e.g., Surface Area), width/length, and interpersonal distances. Reliability: Tracking systems (GPS, LPM, TACTO) showed < 5% error in distance but lower accuracy during high-intensity actions. Tactical Adaptability: Higher irregularity (Shannon Entropy) in spatial distribution was found in smaller spaces and formats with fewer players, indicating greater tactical adaptability.	Practitioners should select SSG constraints based on specific tactical goals; numerical imbalances (e.g., 5 v 4) and smaller pitches are effective for promoting higher exploratory behavior and tactical variability. The choice of tracking variables should align with the desired level of analysis (collective vs. individual) to ensure precise performance monitoring.

De Dios-Álvarez et al. (2021) [32]	Systematic Review	Female soccer players (Under-19 and Seniors), including amateur and professional levels.	Variables: Player numbers, pitch area, game surface, and training regimens.	Synthesized Evidence: Analysis of 13 manuscripts shows a complex interaction between technical, physical, and physiological demands in female soccer. The load profiles are significantly modulated by manipulating player density and pitch dimensions.	SSGs are an effective training resource for female soccer players across different age categories. Practitioners should consciously manipulate constraints (especially player numbers and pitch area) to optimize physiological and physical adaptations. Design must account for the specific needs of U19 versus Senior players to improve the overall soccer training process.

Dello Iacono et al. (2023) [27]	Systematic Review and Meta-analysis	1962 adult soccer players (1876 male, 66 female) aged 17 years or older.	Formats: SSG (2 v 2–4 v 4), MSG (5 v 5–7 v 7), and LSG (8 v 8–10 v 10). Pitch sizes: 15 × 10 m to 90 × 60 m. Variables: Area per player (ApP), length-to-width ratio, and game orientation (score vs. possession).	HSR, VHSR, and sprinting exposure across all formats are significantly lower than in 11-a-side matches (e.g., HSR in matches: 20.2–29.7 m/min vs. SSG: 17.2, MSG: 14.7, LSG: 14.8 m/min). Reliability: Poor intra-individual reliability was found for high-speed measures (pooled CV = 22.8%). Moderators: For every 25 m^2^ increase in ApP, HSR increased by 1.9–2.8 m/min across all formats.	Sided games are not appropriate for fully replicating the high-speed demands of match-play. To reach match-like intensities, practitioners must use larger playing areas (> 200 m^2^/player for HSR and > 365 m^2^/player for sprinting). Since SSGs provide insufficient sprint exposure, they should be supplemented with isolated sprint/conditioning drills to ensure optimal physical overload and hamstring injury prevention.

Fernández-Espínola et al. (2020) [28]	Systematic Review	Youth athletes (U-18) in various team sports, including soccer, basketball, handball, field hockey, and rugby.	Variables: Number of players (1 v 1 to 9 v 9), pitch dimensions (e.g., 15 × 10 m to 90 × 60 m), rule manipulations (touch limits, scoring modes), and coach presence.	Technical Volume: Reducing players (e.g., 4 v 4 to 2 v 2) increased individual technical actions by ~60% in basketball. Pitch Size: Smaller pitches increased passes and dribbles, while larger pitches increased team width and inter-team distances. External Factors: Coach presence was found to decrease the percentage of successful passes due to increased player stress.	SSGs are a primary methodological resource for youth technical-tactical development. Coaches should use smaller formats (≤ 3 v 3) to maximize ball contact and individual skill execution. Larger pitch areas are necessary to foster collective tactical behavior and spatial organization. Rule constraints (e.g., mandatory pass minimums) should be used to transition from direct to positional attacking sequences.

Ferraz et al. (2025) [29]	Systematic Review	1,628 young male football players (U11 to U18 age categories) from 45 studies.	Formats: 1 v 1 to 10 v 10; Pitch area: 37.5–399 m^2^ per player; Objectives: Scoring (GKs or small goals) and ball possession.	Technical: Fewer players promote higher frequency and variability of actions like passes, receptions, and shots. Physical: 3 v 3 and 4 v 4 formats are most effective for optimizing aerobic performance and intensity. Larger pitches significantly increase high-intensity running (HIR) and inter-team distances. Age: Older players (U15–U18) occupy larger areas and adapt better to spatial changes than younger players.	To maximize technical engagement and aerobic load, 3 v 3 and 4 v 4 formats are recommended. Larger pitch dimensions are necessary to simulate match-specific sprinting and high-speed running demands. Design should be age-specific; younger players (U11–U13) may require more structured formats (e.g., GK+4 v 4) to effectively practice tactical principles, such as maintaining possession.

Ferreira-Ruiz et al. (2022) [2]	Systematic Review	Male soccer players from youth to professional levels, synthesized from 47 high-quality studies.	Variables: Imbalanced interactions (numerical superiority), field dimensions, players’ age, number of players (2 v 2 to 9 v 9), and rules (goals, task aim, touch limits).	Pitch Size & Format: Smaller pitches and fewer players significantly increase the frequency of technical-tactical actions and transitions, while larger formats favor longer possessions and collective behaviors. Numerical Imbalance: Attacking teams in superiority expand space (width/depth), while defensive teams in inferiority group closer to their own goal. Age & Rules: Older players exhibit more stable collective behaviors and better width occupation. Rule constraints, such as touch limits, increase interaction frequency but also lead to higher technical error rates.	SSG constraints must be tailored to specific tactical aims. Use smaller formats to maximize individual technical involvement and transition frequency. Larger pitches and formats are superior for training collective organization. Coaches should be aware that rule modifications (e.g., touch limits) may increase interaction density at the cost of execution accuracy.

Hammami et al. (2018) [13]	Systematic Review and Meta-analysis	200 players (186 male, 14 female) from 15 training studies across soccer, handball, rugby, basketball, volleyball, and Australian football.	Formats: 1 v 1 to 7 v 7; Frequency: ~2 sessions/week; Duration: 4–12 weeks; Intensity: Mean HR > 80% HRmax; average blood lactate > 6 mmol/l.	Aerobic: Large beneficial effect on ${\dot{\text{V}}\text{O}}_{2\max}$ (ES = 1.94; p = 0.05) and moderate effect on intermittent endurance (ES = 0.61; p = 0.007). Anaerobic/Speed: Large effect on RSA (ES = -1.19; p = 0.02) and moderate effect on 10/20 m sprints (ES = -0.89; p = 0.03). Technical: Significantly greater improvement in sport-specific skills (e.g., shooting, ball agility) compared to generic or interval training.	SSGs serve as an effective “multicomponent training” strategy, allowing concurrent development of physical fitness and tactical/technical proficiency. Due to high metabolic and cardiovascular stress, SSGs are a time-efficient alternative to HIIT. Designers should prioritize game-specific constraints to maintain high player motivation and enjoyment while ensuring match-specific intensity.

Kunz et al. (2019) [30]	Systematic Review and Meta-analysis	232 young male soccer players (age 13–18; mean 16.2 ± 1.6 years).	Formats: 2 v 1 to 7 v 7; Game durations: 3–4 × 4 min or 2–3 × 6–13 min; Recovery: 1–4 min; varied pitch dimensions.	Endurance: HIIT and SSG showed similar improvements in ${\dot{\text{V}}\text{O}}_{2\mathrm{peak}}$ (ES = 0.15 favoring HIIT), lactate threshold (ES = -0.01), and running economy (ES = 0.05). Neuromuscular: Both HIIT and SSG had trivial impacts on sprinting, jumping, and RSA. Specific Skills: HIIT was superior for COD (ES = 0.59), while SSG was slightly better for soccer-specific movements (Ekblom’s test ES = -0.21).	SSG is a time-efficient alternative to HIIT, allowing simultaneous development of aerobic endurance and tactical/technical skills. However, neither SSG nor typical HIIT (4-min intervals) is sufficient for neuromuscular (sprint/jump) development; separate explosive strength training is required.

Lauria et al. (2024) [31]	Systematic Review	Professional and semiprofessional male soccer players (ages 23–27) from 9 studies (published 2010–2022).	Formats: 2 v 2 to 7 v 7; Relative area: 75–245 m^2^ per player; Manipulations: Ball touch limits (1-touch, 2-touches, 3-touches) vs. free-play.	Physiological: Limiting touches (especially 1-touch) significantly increased blood lactate (LAC) and RPE in 2 v 2, 3 v 3, and 4 v 4 formats compared to free-play. Physical: Generally, touch limits had no significant impact on total distance covered in 6 out of 8 studies. Heart Rate: Discrepancies noted; 6 v 6 formats showed higher %HR in 2-touch vs. free-play, while 7 v 7 showed higher HR in free-play for semi-professionals.	To increase physiological intensity and metabolic strain, practitioners should implement ball-touch limits (1-touch or 2-touches), particularly in small formats (2 v 2 to 4 v 4). However, coaches must be cautious with less experienced players, as strict touch limits can lead to high error frequencies, which may inadvertently reduce the overall physical and physiological load. Design should prioritize touch limits when the goal is to force players to run more frequently at high intensity to create passing lanes.

Maia Junior et al. (2023) [16]	Systematic Review	Male soccer players across 12 studies (Elite, amateur, and university levels; ages 12.1–26.4 years).	Scoring methods: 1 goal (1G), multiple goals (2G, 3G, 4G), and Score Zone (SZ/stop-ball); Formats: 2 v 2 to 8 v 8; Relative area: 19–209 m^2^/player.	Physical: 1G SSGs induce higher internal load (HRmax, TRIMP) than 3G. SZ SSGs elicit higher HR (~90% HRmax) and RPE compared to 1G, 2G, and 3G. Technical/Tactical: 1G increases inaccurate passes and ball losses but favors defensive organization. 2G/3G increases tactical complexity, inter-team distances, and offensive variety.	Coaches should use 1G formats to maximize physiological strain and defensive grouping. SZ (stop-ball) is recommended for high-intensity metabolic conditioning and increasing attacking volume. Multiple-goal configurations are best suited for tactical training to exploit defensive width and improve decision-making under high target density.

McCunn et al. (2021) [33]	Systematic Review	Youth, student, and professional soccer players (Male and Female) from 90 included studies (42 for incidence, 36 for biomechanics, 12 for cognitive).	Formats: 3 v 3 to 5 v 5 SSGs, as well as MSG and LSG formats; Duration: 4–18 min bouts.	Incidence: Heading in SSGs is very low (typically 0–1 header per player per session) compared to matches (1–9 headers/player). Biomechanics: Average head acceleration during heading ranges from 4 to 50 g, which is generally below the injury threshold of < 50 g. Cognitive: 75% of studies (9/12) found no acute negative impact on cognitive performance; 3 studies reported transient effects on reaction time and memory (returning to baseline within 24–48 h).	SSGs are a safe training modality regarding sub-concussive head impacts, as they naturally elicit a very low frequency of heading compared to match play or specific heading drills. Since acute cognitive function is generally preserved, SSGs are suitable for preparation strategies without compromising short-term brain health or decision-making performance.

Milanović et al. (2015) [34]	Systematic Review and Meta-analysis	380 participants (191 male, 189 female; ages 19–76) from 17 studies, including healthy untrained individuals and patients with T2DM, hypertension, or prostate cancer.	Formats: 3 v 3 to 9 v 9; Duration: 40–60 min sessions; Frequency: 2–3 sessions/week; Intensity: 78–84% HRmax (average 82%); Program length: 12–70 weeks.	Aerobic Capacity: Recreational soccer increased ${\dot{\text{V}}\text{O}}_{2\max}$ by an average of 3.51 mL/kg/min. Compared to controls, effects were largely beneficial (ES = 1.46), and moderately better than continuous running (ES = 0.68) and strength training (ES = 1.08). Gender Effects: Improvements were large in men (ES = 1.22) and moderate in women (ES = 0.96). Psychological: Lower RPE compared to HIIT and higher motivation/social interaction compared to running.	Recreational soccer serves as an ideal “Football Fitness” concept for public health, as it naturally elicits high-intensity bouts (> 90% HRmax for ~20% of time) with ~900 activity changes per session. Practitioners should incorporate multiple active breaks during 60-min protocols to enhance work capacity and ${\dot{\text{V}}\text{O}}_{2\max}$ kinetics. The high motivational factor makes SSGs superior to generic running for long-term adherence in sedentary populations and patients with non-communicable diseases.

Moran et al. (2019) [3]	Systematic Review and Meta-analysis	144 male youth soccer players (aged < 18 years) from 7 studies.	Formats: 2 v 2 to 7 v 7; Prescription: ≥ 4 sets of 4-minute bouts; 2 sessions per week; 3-minute recovery periods.	Endurance Performance: Trivial difference between SSG and Conventional Endurance Training (CET) (ES = 0.04; p = 0.86). Both methods significantly improved aerobic performance (SSG ES = 0.82, p = 0.04; CET ES = 0.89, p = 0.04). Moderators: Programs > 8 weeks (ES = 0.45) and sessions with > 4 sets (ES = 0.53) favored SSGs over CET.	SSGs are a time-efficient alternative to CET, enabling concurrent development of endurance and technical skills. Practitioners should use a minimum of 4 sets of 4-minute games with 3-minute rest periods, twice weekly. Longer intervention periods (> 8 weeks) are recommended to ensure SSGs elicit training responses comparable to those elicited by CET, given the unpredictable, acyclic nature of gameplay.

Neag et al. (2024) [35]	Systematic Review and Meta-analysis	Healthy youth soccer players (ages 6–19) across 14 studies (e.g., n=478 for SSG vs. RBM comparisons).	Formats: 1 v 1 to 6 v 6; Duration: 4–8 weeks; Frequency: 2–5 sessions/week; Pitch sizes: 10 × 15 m to 42 × 30 m; Protocols: Intermittent work (30 s to 4 min) with passive/active recovery.	CoDS (Change of Direction Speed): No significant difference between SSG and RBM (ES = -0.04, p = 0.74). Within-group analysis showed significant improvements for both SSG (ES = -0.67, p < 0.001) and RBM (ES = -0.61, p < 0.001). Combined Methods: SSG combined with HIIT, RST, or core strength showed robust improvements (ES = -0.72, p < 0.001).	SSGs are as effective as running-based methods for improving CoDS due to the high density of sport-specific movement changes. To maximize results, practitioners should combine SSGs with HIIT, Repeated Sprint Training (RST), or core strength training to address the limited high-speed running exposure typical of small-sided formats.

Niu et al. (2025) [9]	Pairwise and Network Meta-analysis	914 healthy soccer players from 23 studies (2004–2024).	Formats: 1 v 1 to 7 v 7; Frequency: 2–4 times/week; Period: 3–12 weeks; Compared SSG with SAQ and HIIT.	Sprint: SAQ was significantly more effective than SSG (SMD = -1.03). SSG had the lowest probability of success (SUCRA = 8.1%). COD: HIIT (SUCRA = 67.3%) and SSG (SUCRA = 65.9%) ranked highest in the network analysis, showing better potential for multidirectional agility than SAQ (SUCRA = 63.1%).	SSGs provide insufficient stimulus for maximal linear speed due to frequent directional changes and a lack of standardized acceleration sequences. However, SSGs are highly effective for COD and agility as they enhance perceptual-cognitive factors and decision-making under uncertainty. For optimal development, practitioners should use an integrated approach: SAQ for raw speed and SSGs for game-specific agility and tactical responsiveness.

Ometto et al. (2018) [19]	Systematic Review	365 soccer players (intermediate to advanced/elite levels; ages 11–26) from 24 studies.	Modifications of pitch dimensions (e.g., 28 × 14 m to 62 × 44 m), player numbers (including numerical parity and imbalance/overloading), and scoring targets (size and quantity).	Technical actions: Smaller fields and fewer players significantly increase ball engagement and technical-tactical actions (shots, dribbles, and ball control). Spatial behavior: Larger pitches increase interpersonal distances, effective playing space, and team spread. Tactical behavior: Numerical inferiority promotes penetration principles (moving toward the goal), whereas numerical superiority enhances defensive organization (coverage and concentration). Multiple targets shift play toward lateral corridors.	SSCGs should be used as facilitators to develop tactical intentionality by manipulating constraints to match specific sub-phase goals. To prioritize individual technical skill acquisition and quick decision-making, practitioners should use smaller playing areas with fewer players. Larger formats are recommended for training collective organizations, managing large spaces, and improving team-wide tactical knowledge. Manipulating target numbers (e.g., mini-goals) is an effective strategy to teach field exploitation and lateral movement.

Praça et al. (2022) [10]	Systematic and Meta-Analytical Review	221 male soccer players (U12 to Seniors) competing at regional, national, and international levels.	Formats: 1-a-side to 7-a-side. Relative Area (ApP): Ranging from 25 to 275 m^2^ per player. Variables: Modified via pitch size, number of players, and inclusion of floaters or goalkeepers.	Larger relative areas have significant positive effects on: Total Distance (d = 0.73, medium to large effect), High-Speed Distance (d = 0.93, large effect), and Mean Heart Rate (d = 0.52, medium effect). Higher speeds are achieved in larger areas because transitions require covering more ground.	Relative ApP is a primary determinant of physical and physiological load. Coaches can use ApP calculations to predict and manipulate training intensity across different SSG formats. For instance, if adding floaters decreases the relative area, the pitch size should be proportionally increased to maintain the intended physical load

Rico-González et al. (2022) [4]	Systematic Review	Soccer players (all levels analyzed from a multivariate perspective).	Variables: Match-sized vs. smaller spaces (e.g., 1 v 1), pre-session fatigue protocols, pitch modifications, game principles (defending/formations), and coach instructions.	Spatial Scale: Match-sized spaces improve physical fitness and collective tactical behavior, while micro-structures (1 v 1) enhance individual tactical skills. Creativity: Pre-session exercises that induce fatigue can boost creativity and delay match fatigue. Pedagogy: Differential learning (non-linear pedagogy) promotes improvements across all dimensions, with a major impact on creative thinking.	Practitioners should align spatial constraints with tactical scale: use match-representative dimensions for collective unity and 1 v 1 formats for micro-tactical development. To foster adaptability and creativity, coaches should incorporate differential learning and consider using fatigue-inducing tasks before SSGs to challenge decision-making under stress.

Ridwan et al. (2025) [20]	Systematic Review	Professional and youth male soccer players (U-14 to Seniors).	Formats: 1 v 1 to 6 v 6. Field Sizes: 10 × 15 m to 50 × 31 m. Interventions: Modified rules (with/without boards) and combinations with HIIT or core strength.	Responsiveness: SSGs significantly enhance decision-making and adaptation to dynamic situations. Intensity: 1 v 1 to 3 v 3 formats on compact fields frequently target > 90% HRmax. Variability: 3 v 3 promotes greater within-player heart rate variability, while 5 v 5 is better for stabilizing locomotor demands. Synergy: Combining SSGs with core strength or HIIT markedly improves speed and strength-based conditioning.	To optimize responsiveness and rapid reactions, coaches should implement 1 v 1 to 3 v 3 formats on compact fields (e.g., 10 × 15 m or 12 × 24 m) using brief, high-intensity sets. For tactical endurance, 4 v 4 or 5 v 5 formats are recommended. In high-intensity 1 v 1 games, longer rest intervals are critical to reduce catabolic reactions and the risk of overtraining.

Sarmento et al. (2018) [6]	Systematic Review	77 studies involving elite, professional, and amateur male soccer players across all age categories (U-11 to Seniors).	Variables: Player numbers (1 v 1–10 v 10), pitch area (absolute and relative), goal types (scoring zones, small goals, with/without GK), rule constraints (touch limits, marking styles), and coach encouragement.	Player Number: 1 v 1 to 4 v 4 formats significantly increase physiological demand (HR ~85–90% HRmax) compared to large-sided games (~81% HRmax). Pitch Area: Larger areas (> 100 m^2^/player) increase HR, lactate, and high-speed running distances. Constraints: Limiting touches and man-to-man marking increase internal/external load and technical execution.	Small formats (≤ 4 v 4) are ideal for taxing the glycolytic system and anaerobic capacity but are limited for complex tactical development. Larger pitches are required to develop longitudinal and lateral exploration tactics and higher physical demands. Coaches can use small goals and verbal encouragement to further amplify metabolic intensity.

Silva et al. (2023) [36]	Systematic Review	835 male and female football players (amateur to elite; ages 16–28) from 42 studies.	Formats: 1 v 1 to 10 v 10; varied pitch sizes; specific rules (numerical superiority, touch limits, floaters, and goalkeepers).	Drill Comparisons: SSGs elicit higher ACC and DEC demands than circuit training and other running-based drills. Manipulation: Reducing player numbers or pitch size increases ACC and DEC demands. Positional Demands: Wide players (fullbacks/wide midfielders) typically face higher ACC/DEC demands than central players. Action Frequency: Low-intensity ACC/DEC actions occur more frequently than high-intensity ones.	To maximize mechanical loading (ACC/DEC density), practitioners should prioritize smaller formats or reduced area per player. Design must consider positional specificity, as wide players may require greater physiological preparedness. High ACC/DEC drills should be periodized, with load reduced toward match day (MD-1) and increased during compensatory sessions (MD+1) for non-starters to replicate match-level intensities.

Silva et al. (2023) [7]	Systematic Review	317 male soccer players (aged 16.4–27.6 years) from elite, professional, and youth levels	Microcycle structure: Analysis of external (ACC, DEC, HSR, TD, Sprint) and internal (RPE, %HRmax) loads across “Match Day (MD) minus” formats.	Peak Loading: MD-3 is the most demanding session for ACC (Z=0.89), HSR (Z=0.94), Sprint (Z=1.08), and TD (Z=0.95). MD-4 elicited the highest deceleration demands (Z=0.95). Tapering: MD-1 is consistently the least demanding session for all variables except sprint. Internal Load: MD-3 also showed the highest RPE (Z=1.04) and %HRmax (Z=0.77).	SSGs should be periodized within the microcycle to align with physiological goals. High-intensity “acquisition” sessions, such as 4 v 4, should be scheduled midweek (MD-4/MD-3) to maximize physical overload. MD-1 should serve as a taper, while MD+1 should focus on recovery or compensation for non-starters. Coaches should account for the “wave effect” where load increases toward mid-week and decreases as MD approaches.

Teixeira et al. (2022) [37]	Systematic Review	538 male soccer players (Elite, professional, and youth) from 26 studies.	Formats: Predominantly SSGs (n=17), specifically 5-sided and 6-sided games; Tracking: GPS, LPM, and optical tracking systems (5–18 Hz).	Integration: Positional data effectively integrates physical demands (movement speed, distance within zones, ACC/DEC) with tactical behavior (spatial variability, dyadic synchronization, stretch index, and Voronoi-based areas). Complexity: Nonlinear metrics such as Approximate Entropy (ApEn) and Sample Entropy (SamEn) are used to characterize the predictability and regularity of player movements. Formats: 5 v 5 and 6 v 6 are the most validated formats for concurrent development across the physical, technical, and tactical domains.	Practitioners should adopt integrative spatiotemporal parameters (e.g., synchronization and team centroid) to capture the interplay between physical load and collective tactical outcomes. To ensure accurate tracking of high-intensity, nonlinear movement paths, devices with sampling frequencies > 10 Hz (ideally with embedded accelerometers) are recommended. SSGs should be designed as “repetition without repetition” environments to enhance motor efficiency and decision-making under ecological conditions.

Trotta et al. (2025) [38]	Systematic Review and Meta-analysis	117 highly trained/national-level female soccer players across 4 studies.	Formats: 2 v 2 to 7 v 6; Relative area: 72–110 m^2^ per player; Intensity: 85–90% of VIFT; Bout duration: > 2–3 min.	COD: Significant effect favoring SSGs over HIIT (ES = 0.42; p < 0.001). END: No significant difference (ES = -0.36; p = 0.127). ST: No significant difference (ES = -0.33; p = 0.316). VJ: No significant difference (ES = 0.29; p = 0.126).	Small-format SSGs are superior for enhancing COD in female players due to frequent, rapid directional changes and high-intensity footwork. However, typical relative areas (72–110 m^2^/player) are insufficient for maximal sprint exposure; larger spaces (> 300–365 m^2^/player) or supplementary HIIT and plyometrics are required for linear speed and power development.

Ueda et al. (2023) [5]	Systematic Review	134 soccer players (professional, amateur, U23, U15, and U12 categories) from 5 included studies.	Formats: 4 v 3 to 11 v 11; Relative area: 39.2–336 m^2^/player; Manipulations: Numerical imbalance, goal positioning, and ball types (soccer, handball, rugby).	Creativity & Exploration: Smaller formats (e.g., 5 v 5) lead to more total actions and more original/creative actions than larger formats (7 v 7 or 11 v 11). Numerical Imbalance: Playing with a numerical disadvantage significantly increases exploratory behavior and tactical variability. Structural Constraints: Changing goal positions (e.g., diagonal goals) modifies the originality of tactical behavior. Ball Types: Using non-soccer balls (rugby/handball) tended to decrease fluency and versatility in youth players.	To foster tactical creativity and exploratory behavior, practitioners should prioritize smaller formats with fewer players and reduced pitch dimensions. Numerical imbalances and atypical goal positioning are effective “constraints-led” strategies to provoke original tactical solutions. However, manipulating ball types may not be effective for acute creativity and should be used cautiously within differential learning frameworks.

Zhang et al. (2023) [39]	Systematic Review and Meta-analysis	147 adolescent athletes (10–19 years) in soccer (4 studies), basketball (2), and handball (1).	Formats: 1 v 1 to 9 v 9; Field sizes: 10 × 5 m to 60 × 50 m (soccer) and 15 × 14 m to 30 × 28 m (basketball); Intensity: 80–90% HRmax	LSA: HIIT was significantly better than SSGs (SMD = 0.35; p = 0.007). Both HIIT (SMD = 0.45) and SSGs (SMD = 0.27) significantly improved LSA. RSA: No significant difference between HIIT and SSGs (SMD = -0.05; p = 0.77). HIIT (SMD = 0.36) had a higher effect size than SSGs (SMD = 0.17) in a pre-post comparison.	SSGs are effective for developing RSA and aerobic capacity while concurrently addressing technical/tactical skills. However, to maximize linear speed (LSA), SSGs should be supplemented with HIIT, as SSG intensities may not reach the maximal sprint thresholds required for LSA.

#### Evidence Sources and Coding Structure

For each included systematic review or meta-analysis, the following information was extracted:

– *Review characteristics:* author, publication year, and review type
– *Population characteristics:* age group, competitive level, and general sample characteristics of the primary studies included in the review
– *SSG design characteristics:* player format, pitch dimensions, area per player, bout duration, work-to-rest ratio, and rule manipulations reported in the review
– *Key findings:* reported associations between SSG design variables and performance outcomes, including pooled effect sizes, percentage changes, or narrative summaries when quantitative estimates were not provided
– *Design implications:* statements or interpretations provided by the review that were relevant for informing SSG training design

This structure enabled the development of a structured evidence map summarizing the literature and providing the empirical basis for the synthesis process.

#### Coding of SSG Design Components

To facilitate the synthesis of design principles across reviews, commonly manipulated SSG variables were coded into a set of core design components derived from the intervention descriptions reported in the included reviews. These components represent the most frequently manipulated task constraints used to modify SSG training stimuli.

The following design components were coded:

– *Area per Player* (playing space per player; pitch size manipulation)
– *Format / Number of Players* (2 v 2, 3 v 3, 4 v 4, 6 v 6, etc.; player density)
– *Bout Duration* (length of repetitions or continuous bouts)
– *Work-to-Rest Ratio* (interval structure between work and recovery)
– *Rules / Constraints* (pressing, scoring/target, zonal, conditioning constraints)
– *Touch Limitation* (one-touch, two-touch, limited touches, free touches)
– *Joker Usage* (inside jokers, outside jokers, neutral players; numerical superiority)
– *Directionality & Goalkeeper Involvement* (directional play, goaloriented tasks, presence or absence of goalkeeper)
– *Supplementary Conditioning Strategies* (“top-up” methods added before or after SSG-based drills)

Whenever a review reported how manipulation of these variables influenced training responses, the relationship between the design component and the reported outcome was recorded.

#### Performance Domains

Extracted outcomes were categorized into six performance domains, representing the primary capacities targeted through SSG training:

– *Aerobic and internal load responses* (e.g., heart rate, perceived exertion)
– *Anaerobic and glycolytic demands* (e.g., repeated sprint activity or metabolic stress indicators)
– *High-speed running and sprint exposure*
– *Acceleration–deceleration and change-of-direction demands*
– *Technical execution* (e.g., passes, dribbles, ball contacts)
– *Tactical behaviours* (e.g., team organization, spatial occupation, collective movement patterns)

Both acute responses (physiological and external load indicators) and chronic adaptations (e.g., aerobic capacity or repeated-sprint ability) were extracted when reported.

#### Evidence Synthesis Procedure

The extracted evidence was integrated through a structured qualitative synthesis to identify consistent patterns across the included reviews. First, statements describing relationships between SSG design components and performance outcomes were identified within each review. Second, patterns that appeared across multiple evidence sources were consolidated to identify recurring tendencies linking specific design variables with particular performance responses. Third, when differing findings were reported across reviews, these were interpreted in relation to contextual moderators such as player level, pitch dimensions, or intervention characteristics.

Rather than estimating pooled quantitative effects, the synthesis focused on integrating the available evidence to clarify how different SSG design components influence distinct performance domains. The resulting synthesis provided the conceptual and empirical foundation for the development of the performance-oriented decision matrix, described in the following section.

### Construction of the Decision Matrix

The decision matrix was developed as the applied output of the umbrella synthesis, translating the extracted evidence into a structured framework that links SSG design components to targeted performance outcomes. The matrix was designed to provide practitioners with an integrated overview of how commonly manipulated SSG constraints can be adjusted to elicit specific physical, physiological, technical, or tactical responses.

The construction process followed a structured multi-step procedure based on the evidence summarized in Table 1, which serves as an evidence map of the systematic reviews and meta-analyses included in the umbrella synthesis. First, the six performance domains identified in the synthesis were organized as the matrix rows. These domains included aerobic/internal load responses, anaerobic– glycolytic demands, high-speed running and sprint exposure, acceleration–deceleration and change-of-direction demands, technical execution, and tactical behaviours. All extracted findings from the evidence base were categorized according to these domains to establish a performance-oriented structure.

Second, the core SSG design components were arranged as the matrix columns. These components represent the most frequently manipulated training constraints identified across the literature and include area per player, player format, bout duration, work-to-rest ratio, rule constraints, touch limitations, joker usage, and directionality or goalkeeper involvement. These elements were selected because they consistently emerged across reviews as primary determinants of physiological load, movement demands, and technical–tactical behaviour during SSGs.

Third, each matrix cell was populated by synthesizing the available evidence describing the relationship between a specific design component and a given performance domain. When reviews reported quantitative findings (e.g., effect sizes, percentage changes, or physiological thresholds), these values were used to support the interpretation. In cases where numerical data were not consistently reported, qualitative patterns across reviews were integrated to describe the most commonly observed tendencies.

Finally, the resulting relationships were translated into operational design guidance, allowing each matrix entry to reflect how manipulating a given SSG constraint may influence a specific performance objective. Rather than representing universal prescriptions, the matrix summarizes the most consistent patterns reported in the literature and organizes them into a practitioner-oriented decision framework.

The resulting decision matrix, therefore, serves as the applied synthesis of the umbrella review, providing a structured overview of how SSG design variables can be strategically manipulated to align training tasks with specific performance aims (Figure 2).

**FIG. 2:**
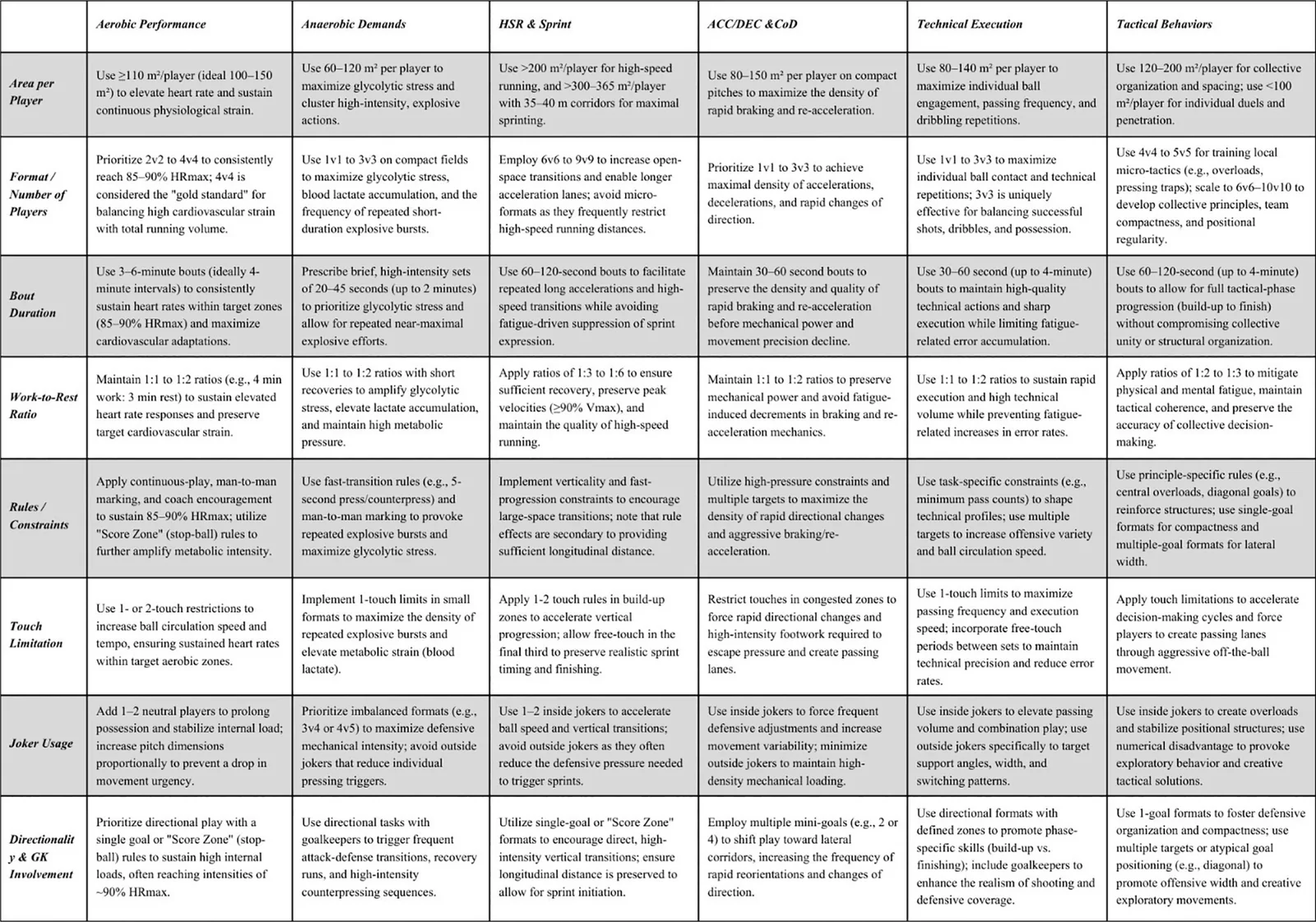
Decision matrix

## RESULTS

As summarized in Table 1, 37 systematic reviews and/or meta-analyses were included in this umbrella synthesis [1–10, 12, 13, 15–39]. The literature search strategy initially identified 286 records, which were reduced to 37 eligible studies after screening. The included reviews reported outcomes across several performance domains associated with SSGs, including aerobic load, anaerobic demands, highspeed running (HSR) and sprinting, neuromuscular/mechanical demands, technical execution, and tactical behaviors [5, 12, 25].

### Aerobic Load Outcomes

As summarized in Table 2, several systematic reviews report that SSG-based training elicits high aerobic internal load responses, often comparable to those observed during running-based high-intensity interval training (HIIT) protocols [13, 17, 23, 37]. Meta-analytic evidence from interventions lasting 3–12 weeks indicates pooled ${\dot{\text{V}}\text{O}}_{2\max}$ improvements of approximately +3.5 mL · kg^−1^ · min^−1^, with mean session intensities typically ranging between ~78–84% HRmax and substantial time spent above 90% HRmax [5, 34]. Similar aerobic responses have been reported across male and female players and across youth and adult populations [3, 32, 34, 37, 38].

**TABLE 2:** Evidence-Based Design Blueprint for Small-Sided Games Targeting Aerobic Load / Aerobic Performance.

Design components	Aerobic Load / Aerobic Performance
**Area per Player** (playing space available per player; pitch size manipulation)	Area per Player (ApP) is a primary determinant of aerobic conditioning and locomotor intensity by regulating movement continuity and defensive density [8, 10, 17]. Synthesized evidence indicates that larger relative pitch areas are consistently associated with moderate-to-large increases in mean heart rate (ES ≈ 0.50–0.52), RPE (ES ≈ 0.70), total distance (ES = 0.73–1.95), and high-speed running (HSR) distance (ES = 0.93–1.20) [6, 8, 10]. Specifically, a dose-response relationship exists where every 25 m^2^ increase in ApP correlates with a 1.9–2.8 m/min rise in HSR exposure [27]. While aerobic protocols typically use reference ranges of 100–150 m^2^ per player, staying above the 100 m^2^ per player threshold is critical for maximizing cardiovascular adaptations [6, 10, 18, 29]. Conversely, smaller dimensions (< 75–100 m^2^) restrict running corridors and shift the stimulus toward mechanical, short-burst actions (e.g., accelerations and decelerations) rather than sustained aerobic flow [23–25, 36].In practice, coaches should prioritize moderate-to-large ApP (~100–150 m^2^ · player−1) for aerobic power, adjusting dimensions upward (> 200 m^2^ · player−1) if concurrent HSR exposure is required, as pitch size manipulation is generally more effective for modulating aerobic intensity than altering player numbers alone [8, 10, 27, 38].

**Format / Number of Players** (team size configuration; e.g., 2 v 2, 3 v 3, 4 v 4, 6 v 6; player density)	Format and the number of players act as fundamental modulators of physiological stimulus, with smaller configurations (1 v 1 to 4 v 4) consistently identified as the most effective for optimizing aerobic performance and cardiovascular intensity [6, 23, 29]. Specifically, formats such as 2 v 2 and 3 v 3 elicit significantly greater metabolic strain, characterized by elevated mean heart rates (~85–90% HRmax), higher blood lactate concentrations, and greater RPE than larger-sided games [6, 18, 20]. While 1 v 1 to 3 v 3 formats on compact fields frequently target intensities exceeding 90% HRmax, the 4 v 4 format is often highlighted as a “gold standard” for balancing high-intensity aerobic strain with greater total running distance [18, 29]. Meta-analytical evidence confirms that SSG interventions using these small formats (e.g., ≥ 4 sets of 4-minute bouts) yield chronic improvements in ${\dot{\text{V}}\text{O}}_{2\max}$ and intermittent endurance comparable to conventional endurance training and running-based HIIT [3, 13, 17, 23, 30, 34]. Conversely, larger formats (5 v 5 to 7 v 7) typically result in reduced physiological intensity (mean HR ~81% HRmax) but are recommended for stabilizing locomotor demands and developing tactical endurance [6, 18, 20]. Consequently, practitioners aiming for maximal aerobic power should prioritize 2 v 2 to 4 v 4 configurations, whereas mid-sized formats should be utilized when the goal is to sustain match-specific running volumes alongside the aerobic load [18].

**Bout Duration** (length of repetitions or continuous play intervals)	Bout duration acts as a critical temporal constraint for regulating cardiovascular intensity and ensuring sustained metabolic strain. Synthesized evidence identifies 4-minute intervals as the “gold standard” for aerobic power development, with protocols utilizing ≥ 4 sets of 4-minute bouts consistently yielding chronic improvements in ${\dot{\text{V}}\text{O}}_{2\max}$ and intermittent endurance comparable to conventional running-based HIIT [3, 23, 24, 30]. While game durations in literature range from 2.5 to 18 minutes [24, 33], shorter, high-intensity bouts (≤ 4 min) are more effective for maintaining heart rates within the target aerobic zone (> 85–90% HRmax) compared to longer, continuous formats, which may lead to a drift in intensity [20, 23, 38]. Furthermore, intervention programs lasting longer than 8 weeks with a high volume of sets (> 4) are associated with superior endurance adaptations [3, 34]. Practitioners should prioritize intermittent regimens (e.g., 4 × 4 min or 10 × 2 min) over continuous play, as these structures allow for higher external load and locomotor intensity without compromising the total aerobic stimulus [17].

**Work-to-Rest Ratio** (ratio between active playing time and recovery time; interval structure]	The work-to-rest ratio serves as a vital modulator, maintaining high-intensity cardiovascular strain while preventing premature neuromuscular fatigue. Intermittent regimens are consistently preferred over continuous play for aerobic development, as these structures allow for higher sustained heart rates and significantly greater external loads without compromising the total metabolic stimulus [17]. Synthesized evidence suggests that recovery periods of approximately 2 to 3 minutes between 4-minute bouts (approximate ratios of 2:1 to 4:3) are highly effective for eliciting chronic improvements in ${\dot{\text{V}}\text{O}}_{2\max}$ and intermittent endurance [3, 23]. Furthermore, incorporating multiple active breaks during training sessions has been shown to enhance work capacity and ${\dot{\text{V}}\text{O}}_{2\max}$ kinetics, making SSGs a more time-efficient alternative to steady-state running [34]. While shorter rest intervals can increase acute metabolic stress, practitioners should ensure adequate recovery to maintain the technical and tactical quality of the game, particularly in high-intensity formats, to avoid excessive catabolic reactions [20].

**Rules / Constraints** (pressing constraints, scoring/target constraints, zonal constraints, conditioning constraints]	Rules and qualitative constraints significantly modulate physiological strain by altering the intensity of player interactions and the urgency of movement. Defensive strategies such as man-to-man marking are consistently shown to increase heart rate, blood lactate, and RPE compared to zone-based defending [6, 17]. Scoring methods also impact the aerobic stimulus; for instance, “Stop-ball” (Score Zone) or single-goal formats often elicit higher cardiovascular strain (e.g., ~90% HRmax) than multi-goal configurations, which may distribute play more laterally and reduce central intensity [8, 16, 19]. Additionally, verbal encouragement from coaches is a proven strategy to amplify metabolic intensity and ensure players maintain the required heart rate zones for aerobic adaptation [6, 28]. While these constraints can increase intensity, practitioners must balance them with the group’s technical skill level to avoid excessive game interruptions that could reduce total aerobic volume [2, 31].

**Touch Limitation** (one-touch, two-touch, limited touches, free touches)	Touch limitations (e.g., one-touch or two-touch) serve as an effective strategic constraint, amplifying physiological intensity and metabolic strain during small-sided games [6, 17, 31]. Synthesized evidence indicates that restricting touches, particularly to a single touch, significantly increases blood lactate concentrations and RPE in small configurations such as 2 v 2, 3 v 3, and 4 v 4 compared to free-play formats [31]. While the impact on heart rate may vary across larger formats, touch constraints generally compel players to move more frequently and with greater urgency to create viable passing lanes, thereby maintaining a high cardiovascular stimulus [6, 31]. However, practitioners should be mindful that strict touch limitations can increase technical error rates and game interruptions, which may inadvertently reduce the total physical load if the game’s flow is compromised [2, 31]. Consequently, to maximize aerobic adaptations, touch limits should be applied to players with sufficient technical proficiency to maintain high, continuous physical intensity aligned with target heart rate zones [2, 31].

**Rules / Constraints** (pressing constraints, scoring/target constraints, zonal constraints, conditioning constraints)	Rules and Constraints (comprising pressing styles, scoring/target methods, zonal restrictions, and conditioning instructions) serve as a major determinant for Tactical Behaviors, as they are the primary strategic tools used to modulate collective organization and the emergence of specific game principles [17, 19, 22]. Scoring targets are a major factor in defining spatial orientation; while single-goal (1G) formats favor defensive grouping and organization, the use of multiple targets (e.g., mini-goals) shifts play toward lateral corridors and encourages players to exploit defensive width [16, 19]. Furthermore, atypical constraints, such as diagonal goal positioning, are major drivers for provoking original tactical solutions and creative exploratory movements [5]. These constraints also play a major role in Aerobic and Internal Load. Implementing man-to-man marking significantly increases physiological demands compared to zonal systems [6, 17]. Scoring modifications are equally impactful; the “Score Zone” (stop-ball) rule elicits significantly higher heart rate (~90% HRmax) and metabolic strain than traditional goal formats [16]. Additionally, external conditioning factors like coach presence and verbal encouragement are major facilitators for amplifying session intensity and maintaining high levels of internal load [6, 28]. Regarding Technical Execution, rules and constraints play a secondary role by influencing the distribution and variety of technical actions; for instance, 1G formats tend to increase inaccurate passes but promote specific skills like shooting and defensive coverage [2, 16, 28]. They also have a secondary impact on External Load (Total Distance and HSR) and Agility/COD. While specific rules like man-to-man marking increase the volume of high-intensity movement, the overall locomotor load remains fundamentally governed by the pitch dimensions and number of players [6, 10, 16, 17]. Finally, the impact of rules and constraints on maximal linear speed and neuromuscular power is negligible, as no rule manipulation can compensate for the lack of longitudinal space and standardized acceleration paths required for maximal velocity development [24, 27, 39].

**Touch Limitation** (one-touch, two-touch, limited touches, free touches)	Touch Limitation is a high-impact design constraint that is a major determinant of Aerobic and Internal Load; research indicates that restricting touches (e.g., 1-touch or 2-touch) significantly increases physiological strain, resulting in higher blood lactate levels and elevated RPE across various formats [6, 31]. It also plays a major role in Technical Execution, as it forces a higher density of technical-tactical interactions and increases the speed of ball circulation, though practitioners should note it often leads to higher technical error rates [2, 28, 31]. In terms of Tactical Behaviors, touch limits are considered major because they demand superior off-the-ball movement and more aggressive creation of passing lanes, which accelerates collective decision-making and transitions [2, 6, 31]. Conversely, touch limitations generally play a secondary role in External Load (Total Distance and HSR); most synthesized evidence indicates that these restrictions do not significantly alter total distance covered compared with free-play, as locomotor load is more heavily determined by pitch dimensions [6, 31]. They also have a secondary impact on Agility and Neuromuscular responsiveness by increasing the frequency of short, sharp movements and rapid reactions required to maintain possession under pressure [6, 20]. Finally, the impact of touch limits on maximal linear speed and vertical power is negligible, as these constraints do not overcome the lack of longitudinal space or the specific mechanics required for maximal velocity adaptations [24, 39].

**Joker Usage** (inside jokers, outside jokers, neutral players; numerical superiority)	Joker usage (neutral players) acts as a strategic modulator of aerobic intensity by altering numerical relations and spatial density. The inclusion of floaters typically creates numerical superiority for the team in possession, facilitating ball retention but may inadvertently reduce the physiological strain on offensive players by decreasing movement urgency and lowering metabolic demands [10, 18, 19]. Crucially, synthesized evidence indicates that adding floaters decreases the relative ApP when pitch dimensions remain constant, thereby reducing mean heart rate and locomotor intensity [10]. Conversely, for the team in numerical inferiority, the defensive task of pressing and covering extra passing lanes can exacerbate cardiovascular strain and exploratory behavior [2, 5, 12]. To optimize aerobic adaptations when utilizing jokers, practitioners should proportionally increase pitch dimensions to preserve the intended ApP, ensuring that numerical superiority does not dilute the targeted cardiorespiratory stimulus [10].

**Directionality & Goalkeeper Involvement** (directional play, goal-oriented tasks, presence or absence of the goalkeeper)	Directionality and the inclusion of goalkeepers serve as critical structural constraints that define the physiological and tactical purpose of the training task. Synthesized evidence indicates that goal-oriented, directional formats—particularly those involving a single goal per team (1G) or “Score Zone” (stop-ball) rules—elicit significantly higher internal loads (HRmax, TRIMP) and RPE compared to possession-based games or configurations with multiple mini-goals [8, 16, 19]. Specifically, “Score Zone” formats are highlighted for their ability to sustain high-intensity metabolic strain, with heart rate responses often reaching ~90% of HRmax [16]. While the presence of goalkeepers can lead to more stable defensive organization, directional play is generally superior for replicating match-specific cardiovascular demands and maintaining movement urgency [17–19, 29]. In contrast, games with multiple targets tend to shift play toward lateral corridors and may distribute the physiological load differently, whereas 1G formats are more effective for maximizing central intensity and defensive grouping [6, 16, 19]. Consequently, practitioners aiming for maximal aerobic power should prioritize directional play with specific scoring targets, utilizing small goals or “Stop-ball” zones to further amplify metabolic intensity and ensure players remain within target aerobic zones [6, 16, 22].

**Top-Up Methods** (applied conditioning add-on strategies)	Top-up methods (applied conditioning add-on strategies) are essential to supplement SSGs, as gameplay alone often provides insufficient exposure to HSR and maximal linear sprinting compared to competitive match demands [24, 25, 27, 39]. While synthesized evidence confirms that SSGs are highly effective for developing aerobic capacity and ${\dot{\text{V}}\text{O}}_{2\max}$—yielding improvements comparable to running-based HIIT or conventional endurance training [3, 13, 17, 23, 30]—they are frequently identified as inadequate for maximal neuromuscular and speed development [9, 17, 24, 30]. Research indicates that “combined training” (integrating SSGs with running-based drills) significantly increases high-intensity running distance (> 19.8 km/h) and physiological strain (blood lactate) compared to SSG-only protocols [15]. Specifically, incorporating intermittent running (e.g., 15s:15s) or isolated sprint/conditioning drills ensures that players reach the necessary mechanical and high-speed thresholds without compromising the game’s tactical or aerobic stimulus [15, 20, 23, 27]. For optimal multi-component adaptations, practitioners should adopt an integrated approach, utilizing SSGs for game-specific agility and cardiorespiratory load while adding supplementary HIIT or sprint sessions to guarantee match-representative locomotor overload [9, 17, 21, 23, 35].

Several reviews report that pitch area per player and player format are associated with differences in cardiovascular responses. Increasing the relative pitch area is consistently associated with higher mean heart rate responses (ES ≈ 0.50–0.52) and higher ratings of perceived exertion (ES ≈ 0.70) [8, 10, 17]. Formats using approximately 100–150 m^2^ per player have been reported to elicit higher cardiovascular responses than smaller pitch areas [6, 10]. In contrast, very small pitch areas (< 75 m^2^) have been associated with greater frequencies of accelerations and mechanical actions and lower sustained cardiovascular responses [16, 20, 34].

Player format also influences internal load responses. Small-sided formats such as 2 v 2 to 4 v 4 have frequently been reported to elicit heart rate responses within the 85–90% HRmax range [6, 18, 20, 29]. While 2 v 2 and 3 v 3 formats often produce the highest relative metabolic strain, several reviews report that the 4 v 4 format is associated with a balance between high-intensity cardiovascular load and greater total running volume [18, 29]. Work-bout duration also influences aerobic responses; several reviews report that intermittent formats with ~4-minute bouts and 2–3-minute recovery periods are commonly used in interventions targeting aerobic power development [3, 23, 24, 30].

Additional task constraints have also been reported to influence cardiovascular responses during SSGs. Rules such as man-to-man marking, coach encouragement, and 1- or 2-touch restrictions have been associated with higher blood lactate concentrations and greater cardiovascular strain than in free-play or zonal formats [6, 17, 31]. Structural constraints, such as directional play with goalkeepers or “Score Zone” rules, have also been reported to increase movement demands, with heart rate responses frequently approaching 90% HRmax [8, 16, 19].

### Anaerobic/Glycolytic and RSA Outcomes

As summarized in Table 3, several systematic reviews report that SSG-based training is associated with improvements in repeatedsprint ability (RSA) and glycolytic-oriented external load, with effects comparable to those observed in running-based HIIT interventions [17, 33, 38]. Meta-analytic evidence from team-sport cohorts reports large improvements in RSA total time (SMD ≈ −1.19) and moderate improvements in 10–20 m sprint performance (SMD ≈ −0.89) following interventions lasting 4–12 weeks [1, 13]. Direct comparisons between SSG and HIIT protocols generally report small and non-significant differences in RSA outcomes (ES ≈ 0.30) [25, 27].

**TABLE 3:** Evidence-Based Design Blueprint for Small-Sided Games Targeting Anaerobic / Glycolytic Demands.

Design components	Anaerobic / Glycolytic Demands
**Area per Player** (playing space available per player; pitch size manipulation)	ApP functions as a critical regulator of anaerobic and glycolytic demands by modulating the density of high-intensity mechanical actions and the frequency of explosive transitions. Synthesized evidence highlights that reducing the relative pitch area significantly increases the volume and density of ACC and DEC, which are primary contributors to the anaerobic load in soccer-specific training [7, 8, 36]. Smaller configurations, particularly those associated with reduced ApP, are consistently linked to significantly higher blood lactate concentrations and greater metabolic strain compared to larger-sided games [6, 18]. While larger relative areas (> 100–150 m^2^ · player−1) facilitate higher peak speeds and total running distance, they tend to decrease the density of short-burst mechanical actions [8, 10]. Conversely, compact dimensions (e.g., 50–100 m^2^ · player−1) maximize the frequency of game-specific anaerobic actions such as tackles, turns, and explosive bursts in congested spaces [18, 20, 36]. To specifically target the glycolytic system, practitioners should utilize these smaller ApP ranges to provoke high-frequency physiological responses, while remaining mindful that extremely restricted spaces (< 50 m^2^ · player−1) may inadvertently limit the locomotor intensity required to reach maximal anaerobic thresholds [24, 25, 27].

**Format / Number of Players** (team size configuration; e.g., 2 v 2, 3 v 3, 4 v 4, 6 v 6; player density)	Format and player density are critical for modulating the contribution of glycolysis and anaerobic metabolism, with smaller configurations (1 v 1 to 3 v 3) consistently eliciting the highest metabolic strain. Meta-analytical evidence confirms that formats smaller than 4 v 4— particularly 2 v 2 and 3 v 3—result in significantly higher blood lactate concentrations and RPE compared to larger-sided games [6, 18]. These small-sided structures, especially when played on compact fields, frequently target intensities exceeding 90% of HRmax, making them ideal for taxing the anaerobic system [6, 20]. While the 4 v 4 format is often cited as a “gold standard” for balancing metabolic and locomotor demands, configurations such as 1 v 1 to 3 v 3 maximize the frequency of explosive, short-burst actions and transitions that drive anaerobic power [2, 18, 36]. Conversely, as player numbers increase (e.g., 6 v 6 and above), the relative glycolytic demand tends to decrease as the game becomes more stable and focused on collective tactical endurance rather than individual anaerobic bursts [6, 18, 22]. Therefore, practitioners seeking to maximize the anaerobic/glycolytic stimulus should prioritize 1 v 1 to 3 v 3 configurations to provoke the high-intensity physiological responses necessary for metabolic adaptation [6, 18, 20].

**Bout Duration** (length of repetitions or continuous play intervals)	Bout duration serves as a critical temporal constraint for modulating glycolytic contribution and ensuring high-intensity anaerobic output. To target maximal anaerobic thresholds (> 90% HRmax) and elevated blood lactate concentrations (> 6 mmol/L), synthesized evidence suggests implementing brief, high-intensity sets ranging from 30 seconds to 2 minutes, particularly in small configurations such as 1 v 1 to 3 v 3 [13, 20, 35]. While 4-minute intervals are standard for aerobic power, shorter work durations (e.g., 45 seconds to 4 minutes) allow players to maintain a higher density of explosive mechanical actions and sport-specific responsiveness without the intensity drift associated with longer, continuous play [20, 23–25, 30]. Research emphasizes that these intermittent structures are superior for taxing the glycolytic system compared to longer durations (e.g., > 6–13 minutes), which tend to shift the metabolic load toward oxidative pathways [20, 30]. Consequently, practitioners should prioritize short, repeated bouts to provoke the necessary metabolic strain for anaerobic adaptations while preventing excessive fatigue-related performance declines [20, 24].

**Work-to-Rest Ratio** (ratio between active playing time and recovery time; interval structure)	The work-to-rest ratio serves as a vital modulator of high-intensity anaerobic output and the accumulation of metabolic byproducts. Intermittent regimens are consistently recommended over continuous play to intensify external load and ensure that players can perform repeated explosive actions [17]. Synthesized evidence indicates that for high-intensity, small-format games (e.g., 1 v 1), longer rest intervals are critical to mitigate excessive catabolic reactions and the risk of overtraining, thereby allowing for the maintenance of maximal effort across sets [20]. While typical aerobic prescriptions utilize 3-minute recovery periods for 4-minute work bouts [3], targeting the glycolytic system through shorter, more intense work intervals requires a recovery structure that preserves the quality of sport-specific responsiveness and decision-making [20]. Consequently, practitioners should balance the work-to-rest ratio to sustain physiological intensities exceeding 90% HRmax and high blood lactate levels (> 6 mmol/L) without inducing premature neuromuscular failure that would compromise the anaerobic training stimulus [13, 20].

**Rules / Constraints** (pressing constraints, scoring/target constraints, zonal constraints, conditioning constraints)	Rules and qualitative constraints significantly modulate the anaerobic and glycolytic profile of small-sided games by altering movement urgency and the density of explosive interactions. Defensive instructions, such as man-to-man marking, are consistently shown to amplify internal load (heart rate, RPE) and metabolic strain (blood lactate) compared to zone-based defending [6, 17]. Scoring methods also play a critical role; specifically, the “Score Zone” (stop-ball) format and single-goal configurations elicit significantly higher heart rates (~90% HRmax) and RPE than multi-goal formats [6, 16]. Furthermore, task constraints that prioritize high-frequency transitions and individual duels in small formats (1 v 1 to 3 v 3) on compact fields are most effective for reaching maximal anaerobic thresholds [2, 20]. The presence of verbal coach encouragement serves as a proven external constraint to ensure exercise intensity remains within target zones, which is particularly vital given the high metabolic stress of anaerobic-focused sessions [6, 20, 28]. While manipulating numerical imbalances (e.g., 5 v 4) can increase exploratory behavior and tactical variability [2, 5, 12], practitioners aiming for maximal anaerobic adaptations should prioritize rules that maximize movement continuity and explosive sport-specific actions.

**Touch Limitation** (one-touch, two-touch, limited touches, free touches)	Touch limitations (e.g., one-touch or two-touch) serve as a potent strategic constraint, amplifying the physiological intensity and metabolic strain required for anaerobic adaptations [6, 17, 31]. Synthesized evidence indicates that restricting touches, particularly in 2 v 2, 3 v 3, and 4 v 4 formats, significantly increases blood lactate concentrations and RPE compared to free-play [6, 31]. These constraints compel players to move with greater urgency and frequency to create viable passing lanes, thereby maintaining a high-intensity metabolic stimulus [6, 31]. However, practitioners should be mindful that strict touch limits often lead to higher technical error rates and game interruptions, which may inadvertently reduce the total physical load if the game’s flow is compromised [2, 31]. To maximize glycolytic power, touch limits should be applied to players with sufficient technical proficiency to maintain a high, continuous density of high-intensity interactions [2, 31].

**Joker Usage (inside jokers, outside jokers, neutral players; numerical superiority)**	Joker usage and numerical imbalances serve as strategic tools for modulating the anaerobic and glycolytic profile of small-sided games by altering movement urgency and defensive density. Synthesized evidence indicates that playing in numerical inferiority significantly increases the physiological and tactical load on the defending team, forcing players to engage in more frequent explosive mechanical actions, such as ACC and DEC, to cover extra passing lanes [5, 26, 36]. While numerical superiority (on the offensive side) often reduces movement urgency and metabolic strain by facilitating ball retention, numerical disadvantage promotes greater exploratory behavior and tactical variability [2, 5, 12]. Crucially, adding a neutral player (joker) without adjusting absolute pitch dimensions reduces the ApP, potentially lowering mean heart rate while increasing the density of short-burst mechanical actions [7, 10]. To maintain the high-intensity physiological stimulus (e.g., > 90% HRmax) required for anaerobic conditioning, practitioners should prioritize imbalanced formats (e.g., 3 v 4 or 4 v 5) to challenge the defensive team or proportionally enlarge the playing area when jokers are included to preserve the intended metabolic load [6, 7, 10, 18].

**Directionality & Goalkeeper Involvement** (directional play, goal-oriented tasks, presence or absence of the goalkeeper)	Directionality and goalkeeper involvement are key structural determinants that shape the anaerobic and glycolytic profile of the training session. Synthesized evidence highlights that goal-oriented, directional formats—specifically those using a single goal (1G) or the “Score Zone” (stop-ball) rule—elicit significantly higher internal loads (HRmax, TRIMP) and RPE than non-directional possession games [8, 16, 19]. The “Score Zone” format is particularly effective for high-intensity metabolic conditioning, often reaching heart rate intensities of ~90% HRmax [16]. While the inclusion of goalkeepers may enhance defensive organization, directional tasks are generally superior for maintaining match-representative movement urgency and explosive anaerobic actions [17–19, 29]. Conversely, multi-goal configurations may increase tactical complexity and lateral movement but can dilute the central metabolic intensity found in 1G or score-zone formats [16, 19]. To optimize glycolytic adaptations, practitioners should prioritize directional play with high-priority scoring targets, such as “Score Zones” or small goals, to ensure consistent physiological strain and high-frequency sport-specific bursts [6, 16, 22].

**Top-Up Methods** (applied conditioning add-on strategies)	Top-up methods (applied conditioning add-on strategies) are critical for targeting anaerobic and glycolytic demands, as game-based training often provides insufficient exposure to maximal linear speed (LSA) and maximal sprinting thresholds [9, 24, 25, 27, 39]. While SSGs are effective for sport-specific agility and repeated-sprint ability, meta-analytical evidence indicates that running-based HIIT and SAQ training are significantly superior for developing raw speed and standardized mechanical overload [9, 17, 24, 39]. Specifically, combined training (integrating SSGs with analytical running) has been shown to elicit higher blood lactate concentrations and greater exposure to very high-speed running (> 19.8 km/h) compared to SSG-only protocols [15, 20]. To ensure comprehensive anaerobic conditioning and injury prevention, practitioners should supplement small-sided formats with isolated sprint or conditioning drills, particularly on “acquisition” days (MD-3/MD-4), to bridge the gap between SSG-induced locomotor load and competitive match demands [7, 15, 17, 27].

Several reviews report that player format and pitch density are associated with differences in glycolytic load during SSGs. Small formats, such as 1 v 1 to 3 v 3, played on relatively compact pitches (area per player ≈ 50–100 m^2^) have been associated with higher blood lactate concentrations (> 6 mmol/L) and higher perceived exertion than larger formats [6, 18, 20]. These formats are also reported to involve a greater frequency of short accelerations, directional changes, tackles, and other explosive actions [18, 36]. Some reviews report that 1 v 1 to 3 v 3 formats frequently elicit heart rate responses above 90% HRmax, whereas formats such as 4 v 4 are associated with a more balanced distribution of metabolic and locomotor load [18, 20].

Work bout duration also influences the metabolic demands of SSGs. Several reviews report that shorter bouts lasting approximately 30 seconds to 2 minutes are commonly used in protocols targeting glycolytic and repeated-sprint demands [13, 20, 35]. Shorter work durations are associated with a higher frequency of explosive mechanical actions, whereas longer continuous formats tend to produce a greater contribution from oxidative metabolism [20, 24, 36]. Rest interval duration has also been reported to influence physiological responses, particularly in very small formats such as 1 v 1 [20].

Additional task constraints may also influence anaerobic load responses. Rule manipulations, such as man-to-man marking and touch restrictions (1- or 2-touch), have been associated with higher blood lactate concentrations and higher ratings of perceived exertion than in free-play conditions [6, 17, 31]. Structural rules such as “Score Zone” (stop-ball) formats have also been reported to increase high-intensity metabolic activity during SSGs [16]. Several reviews also report that SSG formats may provide limited exposure to maximal linear sprinting velocities compared with match play [1, 29, 38].

### High-Speed Running and Sprint Outcomes

As summarized in Table 4, meta-analytic profiling of high-speed running (HSR) and sprinting during SSGs reports mean exposures of approximately HSR ≈ 14.8–17.2 m · min^−1^, VHSR ≈ 2.7–3.6 m · min^−1^, and sprint ≈ 0.2–0.7 m · min^−1^ [7, 27]. These values represent average outputs and show considerable between-session variability (CV ≈ 22.8%), depending on the velocity thresholds applied and the specific task design [27, 40]. Several syntheses consistently report that HSR and sprint distances recorded in SSG, medium-sided games (MSG), and large-sided games (LSG) are lower than those observed during competitive 11-a-side match play [17, 27].

**TABLE 4:** Evidence-Based Design Blueprint for Small-Sided Games Targeting High-Speed Running (HSR) & Sprint Exposure.

Design components	HSR & Sprint Exposure
**Area per Player** (playing space available per player; pitch size manipulation)	ApP is the primary structural determinant for HSR and sprinting exposure, as players require sufficient longitudinal space to reach high-velocity thresholds [15, 24, 27, 29]. Synthesized evidence demonstrates a direct dose-response relationship where every 25 m^2^ increase in ApP correlates with a 1.9–2.8 m/min increase in HSR [27]. Smaller relative areas (e.g., < 75–100 m^2^/player) are consistently shown to restrict sprinting demands and provide insufficient stimulus for maximal linear speed development [24, 25, 38]. To replicate match-representative HSR intensities, pitch dimensions must be significantly enlarged to exceed 200 m^2^/player, while reaching maximal sprinting thresholds (sprints > 25.1–25.2 km/h) requires areas greater than 300–365 m^2^/player [8, 10, 27, 38]. Larger pitches lead to significantly greater high-speed distances (ES = 0.93–1.20) because transitions and collective team spread allow for covering more ground at higher velocities [8, 10]. Consequently, practitioners aiming for HSR and sprint overload should prioritize these very large dimensions, acknowledging that traditional small-format configurations on compact fields are inherently limited for these specific locomotor demands [10, 17, 27].

**Format / Number of Players** (team size configuration; e.g., 2 v 2, 3 v 3, 4 v 4, 6 v 6; player density)	Format and team size configuration play a secondary but significant role in pitch dimensions, influencing locomotor intensity, with mid- to large formats generally offering better conditions for HSR than micro formats. Synthesized evidence indicates that while small configurations (2 v 2, 3 v 3) are superior for metabolic and technical strain, they often restrict high-speed distances due to limited longitudinal space and frequent game interruptions [6, 17, 18]. Specifically, the 4 v 4 format has been identified as a potential “threshold” for locomotor optimization, covering significantly more total distance and distance at speeds > 18 km/h than 2 v 2 configurations [18]. As player numbers increase toward medium-sided (5 v 5–7 v 7) and large-sided games (8 v 8–10 v 10), the collective team spread and passing sequence lengths increase, which can facilitate more sustained running bouts [2, 19, 22]. However, meta-analytical data reveal that HSR and sprinting exposure across all common formats—SSG (2 v 2–4 v 4), MSG (5 v 5–7 v 7), and LSG (8 v 8–10 v 10)—remain consistently lower than the demands of 11-a-side competitive matches [27]. Consequently, while 4 v 4 to 6 v 6 formats are more effective at stabilizing locomotor demands than 2 v 2, practitioners must ensure that player density remains low enough (high ApP) to allow players to reach target velocity thresholds, regardless of team size [18, 27, 29].

**Bout Duration** (length of repetitions or continuous play intervals)	Bout duration serves as a temporal constraint on locomotor output, with synthesized evidence typically using intervals ranging from 2.5 to 4 minutes [6, 17, 24]. Intermittent regimens (e.g., brief, high-intensity sets) are consistently preferred over continuous play to increase external load and ensure that players can maintain the required intensity for high-speed running actions [17, 20]. However, research consistently shows that, regardless of duration or format (SSG, MSG, or LSG), HSR and sprinting exposure in sided games remain significantly lower than those of competitive 11-a-side match demands [27]. The high variability (CV = 22.8%) of high-speed measures across bouts suggests that longer durations might further decrease the likelihood of achieving target sprinting thresholds [27]. Consequently, practitioners should employ brief, high-intensity repetitions to maximize locomotor potential while acknowledging the critical need for supplementary sprint-specific training (e.g., LSA or HIIT) to bridge the gap with match-level demands [24, 27, 39].

**Work-to-Rest Ratio** (ratio between active playing time and recovery time; interval structure)	The work-to-rest ratio is a critical factor in maintaining the quality of high-speed actions and preventing neuromuscular fatigue [17, 20]. Synthesized evidence consistently indicates that intermittent regimens (work sets separated by rest) are superior to continuous play for maximizing high-speed running distances and external load [17]. While 3-minute recovery periods are commonly utilized for 4-minute aerobic-focused bouts [3], targeting maximal locomotor outputs requires ensuring players are sufficiently recovered to perform repeated explosive actions [20]. Specifically, in high-intensity formats such as 1 v 1, longer rest intervals are recommended to mitigate catabolic stress and maintain the intensity required for near-maximal velocity thresholds [20]. Despite optimized recovery structures, practitioners should acknowledge that SSG-based sprint exposure remains significantly lower than match demands, underscoring the importance of recovery management to preserve the quality of the limited high-speed stimulus available [17, 24, 27].

**Rules / Constraints** (pressing constraints, scoring/target constraints, zonal constraints, conditioning constraints)	Rules and qualitative constraints significantly modulate locomotor output by influencing movement urgency and spatial dynamics. Synthesized evidence indicates that directional play and specific scoring formats, such as the “Score Zone” (stop-ball) rule, elicit significantly higher physiological intensities (~90% HRmax) and RPE than standard goal-oriented or possession tasks [16, 19]. Defensive constraints, such as man-to-man marking, consistently increase both internal and external loads compared to zonal defending, as they demand continuous high-intensity tracking and reactive bursts [6, 17]. While touch limitations (e.g., 1-touch or 2-touch) are often implemented to force players to run more frequently at high intensities to create passing lanes, they may not significantly increase total distance and can lead to higher technical error rates [2, 31]. Despite these tactical manipulations, exposure to HSR and sprinting across various rule-based constraints remains significantly lower than match-play demands [17, 27]. Therefore, practitioners should prioritize constraints that promote explosive transitions and high-priority scoring targets to maximize the available locomotor stimulus within the game [2, 6, 16].

**Touch Limitation** (one-touch, two-touch, limited touches, free touches)	Touch limitations (e.g., one-touch or two-touch) are used as strategic constraints to increase movement urgency and the density of technical-tactical interactions. Synthesized evidence indicates that restricting touches, particularly in 2 v 2 to 4 v 4 formats, significantly amplifies physiological intensity (blood lactate and RPE) compared to free-play [6, 31]. While these constraints are intended to force players to run more frequently at high intensities to create passing lanes, research has shown that they often have no significant impact on total distance covered [31]. Furthermore, strict touch limits can lead to a higher frequency of technical errors and game interruptions, which may counteract the intended locomotor stimulus if the game’s flow is compromised [2, 31]. Because HSR and sprinting exposure in all-sided game formats remain substantially below match-play demands regardless of touch rules, practitioners should use touch limits primarily to enhance metabolic strain and technical-tactical responsiveness rather than as a primary tool for achieving maximal velocity thresholds [2, 27, 31].

**Joker Usage** (inside jokers, outside jokers, neutral players; numerical superiority)	Joker usage and numerical imbalances (e.g., 4 v 4 + 2 floaters) are strategic manipulations that alter tactical density and movement urgency. Synthesized evidence indicates that adding floaters/jokers typically reduces the ApP when pitch dimensions remain constant, thereby potentially decreasing physical and physiological load, including HSR distance [7, 10]. While numerical superiority (attacking side) facilitates easier ball retention and positional regularity, numerical inferiority (defending side) forces players to cover more ground and engage in higher exploratory behavior, though often through mechanical actions rather than high-velocity sprints [2, 5, 12, 19]. To preserve the HSR and sprint stimulus when using jokers, practitioners must proportionally increase the absolute pitch size to maintain high ApP thresholds—specifically exceeding 200 m^2^/player for HSR and 365 m^2^/player for sprinting [10, 27]. Without these spatial adjustments, the inclusion of neutral players tends to favor technical-tactical density over the longitudinal distance required to reach maximal velocity thresholds [2, 10, 27].

**Directionality & Goalkeeper Involvement** (directional play, goal-oriented tasks, presence or absence of the goalkeeper)	Directionality and scoring targets are structural constraints that shape the locomotor profile by defining the orientation and depth of play. Synthesized evidence indicates that directional, goal-oriented formats—specifically the “Score Zone” (stop-ball) rule—elicit significantly higher physiological intensities (~90% HRmax) and RPE compared to non-directional possession games [8, 16, 19]. While the inclusion of goalkeepers can enhance defensive organization and tactical realism, the primary driver for HSR remains the availability of longitudinal space to facilitate transitions [10, 19]. Research highlights that multi-goal configurations tend to shift movement toward lateral corridors, whereas single-goal or “Score Zone” formats encourage more direct, high-intensity vertical transitions [16, 19]. Despite these manipulations, HSR and sprinting exposure in directional SSGs consistently fall below match-play levels [17, 27]. Therefore, practitioners should prioritize directional tasks that maximize transition distance and use “Score Zones” to maintain high movement urgency, while acknowledging that goalkeepers may slightly reduce player density and absolute running volumes if pitch dimensions are not adequately enlarged [10, 16, 27].

**Top-Up Methods** (applied conditioning add-on strategies)	Top-up methods (applied conditioning add-on strategies) are essential for HSR and sprint development, as SSGs consistently provide lower exposure to maximal linear speed and sprinting thresholds than competitive matches [9, 24, 27, 39]. Meta-analytical evidence confirms that running-based HIIT and SAQ training are significantly more effective than SSG-only protocols for improving raw speed and neuromuscular power [9, 24, 39]. Specifically, combined training (integrating SSGs with analytical running or intermittent runs) has been shown to increase the total distance covered at high velocities (> 19.8 km/h) and provide a more standardized mechanical stimulus [15, 23, 27]. To ensure comprehensive conditioning and bridge the locomotor gap, practitioners should supplement game-based sessions with isolated sprint drills and plyometrics, particularly to meet the velocity requirements necessary for performance and injury prevention [17, 24, 38].

Several reviews report that area per player is associated with differences in HSR exposure during SSGs [8, 10, 17]. A dose-response relationship has been reported, with approximately every 25 m^2^ increase in area per player corresponding to an increase of ~1.9–2.8 m · min^−1^ in HSR exposure across formats [27]. Studies also report that larger pitch areas (> 200 m^2^ per player) are associated with higher match-like HSR exposures [8, 10, 27]. Achieving maximal sprint speeds (> 25.1–25.2 km · h^−1^) during gamebased training is less frequently reported and has been associated with very large pitch dimensions (> 300–365 m^2^ per player) and the presence of longer running corridors [8, 27, 38]. In contrast, compact formats with smaller pitch areas (< 75–100 m^2^ per player) are generally associated with lower exposure to high-velocity running [24, 25, 38].

Player format also influences locomotor outputs. Larger formats (≥ 6 v 6) are generally associated with greater opportunities for longer accelerations and open-space transitions compared with microformats [6, 39]. Some reviews report that formats such as 4 v 4 produce greater distances at speeds > 18 km · h^−1^ compared with smaller formats such as 2 v 2 [18]. Rule manipulations may also influence locomotor responses; directional play with goalkeepers, manto-man marking, and “Score Zone” (stop-ball) formats have been reported to produce higher volumes of high-intensity running than non-directional possession formats [6, 16, 17, 19].

Several syntheses also report that SSG formats often provide lower exposure to maximal sprinting speeds compared with competitive match play [25, 27]. Consequently, some studies describe the use of additional running drills such as linear sprints, repeated-sprint exercises, or running-based HIIT sessions alongside SSG training [7, 9, 15, 24, 27, 29, 39].

### Acceleration–Deceleration and Change-of-Direction Outcomes

As summarized in Table 5, several reviews report that smaller formats (1 v 1 to 3 v 3) and reduced pitch areas per player (≤ 75–100 m^2^) are associated with higher frequencies of accelerations (ACC), decelerations (DEC), and changes of direction (CoD) during SSGs [17, 18, 36, 38]. In contrast to high-speed running exposures, increasing pitch size (> 250 m^2^ per player) has not been consistently associated with higher volumes of explosive mechanical actions [8, 10]. Compact pitches and small formats are therefore frequently reported to produce higher densities of short accelerations, braking actions, and multidirectional movements [20, 36, 38].

**TABLE 5:** Evidence-Based Design Blueprint for Small-Sided Games Targeting Acceleration–Deceleration (ACC–DEC) & Change of Direction (CoD).

Design components	ACC–DEC & CoD
**Area per Player** (playing space available per player; pitch size manipulation)	ApP and pitch dimensions are the primary regulators of mechanical load density, specifically regarding ACC and DEC frequency. Synthesized evidence indicates that reducing player numbers or pitch size directly increases ACC and DEC demands, making smaller spaces more effective for targeting mechanical overload [36]. Compact fields (e.g., 10 × 15 m to 12 × 24 m) and smaller formats (1 v 1 to 3 v 3) are superior for promoting high-intensity footwork and rapid CoD, which are critical for sport-specific agility [20, 38]. Unlike HSR, which requires large longitudinal distances, meta-analytical data show that increasing pitch size (> 250 m^2^/player) does not significantly increase ACC/DEC actions compared to smaller configurations [8, 10]. Therefore, to maximize the frequency of explosive mechanical bursts and individual technical-tactical transitions, practitioners should prioritize reduced ApP and smaller playing areas [2, 19].

**Format / Number of Players** (team size configuration; e.g., 2 v 2, 3 v 3, 4 v 4, 6 v 6; player density)	Format and team size are the primary regulators of mechanical load density, as smaller configurations (1 v 1 to 3 v 3) provide the most potent stimulus for ACC, DEC, and CoD [20, 36, 38]. Reducing the number of players directly increases the frequency of explosive mechanical actions and individual technical-tactical transitions [18, 36]. Specifically, micro-formats (1 v 1 to 3 v 3) on compact pitches are superior at promoting high-intensity footwork and rapid CoD, effectively targeting game-specific agility and neuromuscular responsiveness [9, 20, 38]. While some evidence suggests that total ACC/DEC counts may be similar across formats when normalized, the density and frequency of high-intensity mechanical bursts and individual ball engagement are markedly higher in smaller formats compared to larger-sided games [2, 6, 18]. Consequently, practitioners should prioritize 1 v 1 to 3 v 3 formats to maximize neuromuscular loading and simulate the high-frequency mechanical demands found in competitive match-play [18, 20, 36].

**Bout Duration** (length of repetitions or continuous play intervals)	Bout duration acts as a critical temporal constraint for regulating mechanical load density and ensuring the quality of explosive actions such as ACC, DEC, and CoD [17, 20]. Synthesized evidence typically utilizes intervals ranging from 2.5 to 4 minutes to facilitate high-intensity performance [6, 17, 24]. To maximize mechanical load accumulation and the frequency of explosive bursts, intermittent regimens are consistently preferred over continuous play [17, 20]. Specifically, the implementation of brief, high-intensity repetitions (ranging from 30 seconds to 4 minutes) in micro-formats is highly effective for enhancing game-specific responsiveness and CoD speed by increasing the density of rapid movement changes [20, 35]. Maintaining shorter bout durations is essential to avoid mechanical power degradation and to ensure that each repetition delivers a maximal neuromuscular stimulus [20].

**Work-to-Rest Ratio** (ratio between active playing time and recovery time; interval structure)	The work-to-rest ratio is a primary determinant of preserving the quality of high-intensity mechanical actions and managing neuromuscular fatigue [17, 20]. Synthesized evidence suggests that intermittent regimens (e.g., 4-minute work sets with 3-minute recovery) are significantly more effective than continuous play for maintaining acceleration density and external load [3, 17]. For high-intensity micro-formats such as 1 v 1 to 3 v 3, implementing longer rest intervals is critical to mitigate catabolic stress and ensure that players can perform repeated explosive CoD without the performance decay associated with metabolic fatigue [20]. Proper recovery management enables greater accumulation of high-intensity ACC and DEC across multiple bouts, which is essential for developing game-specific responsiveness and mechanical power [17, 20].

**Rules / Constraints** (pressing constraints, scoring/target constraints, zonal constraints, conditioning constraints)	Structural and qualitative constraints are essential for modulating mechanical density by forcing rapid adjustments in movement speed and direction [19, 36]. Synthesized evidence highlights that man-to-man marking significantly increases both internal and external loads compared to zonal defending, as it demands continuous high-intensity tracking and reactive bursts [6, 17]. Numerical imbalances (e.g., 4 v 5) also influence movement dynamics; numerical inferiority forces players to increase exploratory behavior and defensive tracking frequency, although often through high-density mechanical actions rather than linear sprinting [2, 5, 12, 19]. Scoring constraints, particularly the “Score Zone” (stop-ball) rule, are recommended for high-intensity metabolic conditioning and increasing attacking volume compared to standard goals [6, 16]. Furthermore, utilizing multiple targets shifts play toward lateral corridors, effectively increasing the frequency of CoD to exploit defensive width [16, 19]. While touch limitations (e.g., 1-touch or 2-touch) increase physiological intensity and movement urgency, they may not significantly impact total distance and can lead to higher technical error rates [2, 6, 31].

**Touch Limitation** (one-touch, two-touch, limited touches, free touches)	Touch limitations (e.g., one-touch or two-touch) are strategic constraints used to increase movement urgency and the density of explosive technical-tactical interactions [2, 31]. Synthesized evidence indicates that restricting touches significantly amplifies physiological intensity (RPE and blood lactate) and increases external load demands compared to free-play [6, 31]. These constraints force players to engage in more frequent high-intensity bursts to create passing lanes and escape pressure, thereby potentially increasing the density of mechanical actions [17, 31]. However, coaches should be cautious, as strict touch limits can increase the frequency of technical errors and game interruptions, potentially compromising the fluidity of mechanical loading [2, 31]. Consequently, touch limitations should be used to modulate metabolic strain and technical responsiveness while maintaining the quality of ACC and DEC through appropriate skill levels [2, 6, 31].

**Joker Usage** (inside jokers, outside jokers, neutral players; numerical superiority)	Joker usage and numerical imbalances (e.g., 4 v 4 + 2 floaters) are strategic manipulations that significantly alter tactical density and mechanical movement urgency [2, 12]. Synthesized evidence indicates that playing with a numerical disadvantage (inferiority) promotes higher exploratory behavior and increases the frequency of defensive tracking and reactive bursts, effectively targeting ACC and DEC density [2, 5, 12, 19]. Conversely, numerical superiority (on the attacking side) facilitates positional regularity and easier ball retention, which may reduce individual mechanical load if movement urgency decreases [19]. Practitioners must be cautious: adding jokers or floaters to a fixed pitch size reduces the ApP, which can inadvertently decrease the overall physical and physiological load [10]. To maintain high-intensity mechanical demands when using neutral players, practitioners should use small formats and adjust pitch dimensions to prevent excessive congestion that could limit explosive footwork and rapid changes of direction [2, 10, 20].

**Directionality & Goalkeeper Involvement** (directional play, goal-oriented tasks, presence or absence of the goalkeeper)	Directionality and goalkeeper involvement significantly influence mechanical load by defining the spatial orientation and tactical urgency of the game [16, 17, 19]. Goal-oriented tasks, particularly the “Score Zone” (stop-ball) rule, are highly effective for increasing movement urgency and physiological intensity compared to standard goal formats [6, 16]. While the inclusion of goalkeepers enhances tactical realism and defensive organization, their presence can reduce the relative ApP, potentially lowering mechanical density if pitch dimensions are not adjusted [10]. To maximize the frequency of CoD, practitioners should use multiple target configurations (e.g., 2 or 4 mini-goals), as these shifts push players toward lateral corridors and force them to frequently reorient their movement to exploit defensive width [16, 19]. Furthermore, manipulating goal positioning (e.g., diagonal or atypical placements) serves as a potent constraint to provoke original tactical solutions and varied mechanical movement patterns [5].

**Top-Up Methods** (applied conditioning add-on strategies)	Top-up methods are essential because SSGs, while effective for game-specific agility, often provide insufficient or inconsistent stimuli for maximal linear speed and explosive neuromuscular power [9, 17, 24, 27, 30]. Synthesized evidence indicates that combined training protocols—integrating SSGs with running-based HIIT, Repeated Sprint Training, or core strength—result in robust improvements in CoD speed and overall mechanical responsiveness [20, 35]. Specifically, SAQ training is significantly more effective than SSG-only protocols for developing raw linear speed, whereas SSGs are superior for enhancing perceptual-cognitive agility and decision-making [9]. To ensure comprehensive mechanical loading and injury prevention (e.g., hamstring health), practitioners should supplement game-based sessions with isolated sprint drills and plyometrics, particularly to reach match-play sprint thresholds [24, 27]. Furthermore, combining SSGs with intermittent runs helps standardize locomotor overload while maintaining tactical specificity [15, 23].

Meta-analytic evidence also reports that SSG-based interventions are associated with improvements in change-of-direction performance comparable to those observed following running-based HIIT or speed– agility–quickness (SAQ) training [33–35]. In youth and female soccer cohorts, SSG interventions have been associated with improvements in multidirectional agility, with reported effect sizes ranging from ES ≈ 0.42 to 0.67 [9, 35, 38]. Work-bout duration has also been reported to influence mechanical load responses. Several reviews describe the use of short bouts lasting approximately 30–90 seconds combined with brief recovery intervals (e.g., 1:1 to 1:2 workto-rest ratios), which are associated with high frequencies of braking and re-acceleration actions [13, 20, 38].

Task constraints have also been reported to influence ACC–DEC density. Defensive instructions, such as man-to-man marking, and rule manipulations, such as “Score Zone” (stop-ball) formats, have been associated with higher acceleration–deceleration frequencies compared with zonal or traditional goal-oriented formats [6, 16, 17]. The use of multiple targets (e.g., two or four mini-goals) has also been reported to increase the frequency of lateral movements and directional changes during play [16, 19]. Several studies describing weekly training structures report that higher mechanical loads from SSG formats are often scheduled earlier in the weekly microcycle (e.g., MD-4 or MD-3), with reduced loading closer to competition (e.g., MD-1) [7]. Some studies also describe the use of additional mechanical drills alongside SSG formats to increase exposure to accelerations and decelerations during training [9, 29, 38].

### Technical Execution Outcomes

As summarized in Table 6, several reviews report that smaller SSG formats (1 v 1 to 4 v 4) and reduced playing space are associated with greater individual on-ball involvement during training tasks [1, 9, 24, 28]. Smaller areas per player and fewer players have been associated with higher densities of technical actions. For example, reducing format size from 4 v 4 to 2 v 2 has been reported to increase the number of individual technical actions by approximately 60% [2, 19, 28]. Some reviews also report that 3 v 3 formats are frequently associated with high rates of technical actions, such as shots, dribbles, and ball possessions [18, 29].

**TABLE 6:** Evidence-Based Design Blueprint for Small-Sided Games Targeting Technical Execution.

Design components	Technical Execution (passing, ball control, dribbling, error rate)
**Area per Player** (playing space available per player; pitch size manipulation)	ApP and pitch dimensions are primary determinants of technical engagement and action density [8, 19]. Synthesized evidence demonstrates that reducing the playing area significantly increases the frequency and variability of technical actions, including shots, dribbles, ball control, and successful passes [2, 19, 29]. Specifically, smaller pitches promote greater individual ball engagement and more frequent technical-tactical transitions than larger, match-representative spaces [2, 19, 28]. While larger areas are necessary to foster team width and longer passing sequences, smaller ApP configurations are superior for maximizing technical volume and executing skills under high-pressure conditions [6, 8, 28]. Therefore, to prioritize individual skill acquisition and high-frequency technical interactions, practitioners should utilize reduced pitch sizes and compact formats [1, 19, 28, 29].

**Format / Number of Players** (team size configuration; e.g., 2 v 2, 3 v 3, 4 v 4, 6 v 6; player density)	Format and team size are critical determinants of technical load, with smaller configurations (1 v 1 to 3 v 3) maximizing the frequency and variability of technical actions [1, 28, 29]. Reducing player numbers (e.g., from 4 v 4 to 2 v 2) significantly increases individual ball engagement and technical-tactical transitions compared to larger formats [2, 19, 28]. Specifically, the 3 v 3 format is identified as uniquely effective for technical development, providing an optimal balance for successful shots, dribbles, and ball possession [18, 29]. Furthermore, small formats (≤ 3 v 3) are highly recommended to maximize ball contact and provide the diverse stimuli necessary for rapid technical skill acquisition [1, 28]. While larger formats (e.g., 5 v 5+) are better suited for training collective team organization, micro-formats are superior for prioritizing individual duels and skill execution under game-specific pressure [2, 19, 22].

**Bout Duration** (length of repetitions or continuous play intervals)	Bout duration is a critical factor in maintaining the quality and precision of technical execution, as it regulates fatigue accumulation that can impair skill performance [6, 17, 20]. Synthesized evidence indicates that typical intervals for technical-focused SSGs range from 2.5 to 4 minutes per set [6, 23, 24]. Intermittent regimens consisting of multiple brief sets (e.g., 4 sets of 4 minutes) are superior to continuous play for preserving a high volume of successful technical actions, such as passing and ball control, by providing adequate recovery periods between bouts [3, 6, 17]. Specifically, short, high-intensity bouts in micro-formats (ranging from 30 seconds to 4 minutes) are recommended to maximize individual ball engagement while ensuring technical execution remains sharp and cognitively responsive [20, 28]. Maintaining shorter durations prevents the degradation of technical accuracy often associated with high metabolic strain [20].

**Work-to-Rest Ratio** (ratio between active playing time and recovery time; interval structure)	The work-to-rest ratio is a key determinant of maintaining the precision and volume of technical actions by managing player fatigue and recovery [17, 20]. Implementing intermittent structures, such as protocols using at least 4 sets of 4-minute games with 3-minute recovery periods, is effective for ensuring that players sustain high levels of technical engagement and accuracy throughout a session [3]. For high-intensity micro-formats (e.g., 1 v 1 to 3 v 3), longer rest intervals are particularly critical to mitigate catabolic stress and prevent performance decay, which can lead to technical errors and decreased responsiveness [20]. Furthermore, incorporating multiple active breaks within longer protocols enhances overall technical work capacity, ensuring that skill execution remains sharp and effective under game-specific pressure [20, 34].

**Rules / Constraints** (pressing constraints, scoring/target constraints, zonal constraints, conditioning constraints)	Structural and rule-based constraints are strategically used to modulate technical intensity and the frequency of individual interactions [5, 19, 36]. Man-to-man marking, compared with zonal defense, significantly increases both the internal/external loads and the technical demands on players [6, 17]. Manipulating scoring targets, such as using multiple mini-goals, is an effective strategy to encourage offensive variety and exploit defensive width [16, 19]. The implementation of a “Score Zone” (stop-ball) rule is specifically recommended to increase attacking volume and metabolic strain compared with traditional goals [6, 16]. Numerical imbalances (e.g., attacking superiority) force distinct tactical adaptations; attacking teams in superiority tend to expand the playing space (width/depth), while defensive teams in inferiority focus on grouping and coverage [2, 19]. Furthermore, touch limitations (e.g., 1-touch or 2-touch) amplify technical-tactical interaction density and movement urgency, though practitioners should note that they often lead to higher technical error rates, especially among less experienced players [2, 6, 31].

**Touch Limitation** (one-touch, two-touch, limited touches, free touches)	Touch limitations (e.g., one-touch or two-touch) are strategic constraints used to increase the density and urgency of technical-tactical interactions [2, 31]. Evidence suggests that restricting touches significantly amplifies physiological intensity and internal load compared to free-play [6, 31]. These constraints force players to engage in more frequent technical actions under pressure, thereby increasing interaction frequency and decision-making speed [2, 17]. However, practitioners must be cautious as strict touch limits often lead to higher technical error rates and reduced execution accuracy, particularly in less experienced or youth players [2, 28, 31]. Therefore, touch limitations should be carefully balanced with the players’ skill levels to ensure that technical quality is not compromised by excessive task complexity [2, 31].

**Joker Usage** (inside jokers, outside jokers, neutral players; numerical superiority)	Synthesized evidence indicates that teams in numerical superiority (attacking side) tend to expand the playing area in both width and depth, which facilitates positional regularity and successful ball retention [2, 19]. Conversely, playing in numerical inferiority (on the defensive side) promotes greater exploratory behavior and tactical variability, forcing players to adapt their technical solutions under disadvantageous conditions [5, 12]. It is important to note that adding neutral players to a fixed pitch size reduces the relative ApP, potentially increasing technical congestion; therefore, pitch dimensions should be adjusted proportionally to maintain the desired technical load [10]. Smaller formats featuring jokers are particularly effective for fostering individual duels and increasing the frequency of successful offensive transitions [2, 18].

**Directionality & Goalkeeper Involvement** (directional play, goal-oriented tasks, presence or absence of the goalkeeper)	Directionality and goalkeeper involvement are key determinants of technical focus, as they define the spatial orientation and tactical purpose of the game [16, 19]. Goal-oriented tasks, particularly the “Score Zone” (stop-ball) rule, are recommended to increase attacking volume and individual technical engagement compared with standard goal formats [16]. Utilizing multiple targets (e.g., 2 or 4 mini-goals) effectively shifts play toward lateral corridors, encouraging players to exploit defensive width and increase the variety of passing and dribbling solutions [16, 19]. While the inclusion of goalkeepers enhances tactical realism, their presence can reduce the relative ApP, which may increase technical congestion; therefore, pitch dimensions should be adjusted to maintain the desired space for skill execution [10, 19]. Furthermore, manipulating goal positioning, such as diagonal or atypical placements, serves as a potent constraint that provokes original tactical behaviors and creative technical executions [5].

**Top-Up Methods** (applied conditioning add-on strategies)	Top-up methods are recommended to ensure balanced development, as technical actions within SSGs often exhibit high variability and may not reach the standardized thresholds required for maximal mechanical adaptation [17, 21, 27]. While SSGs are superior to generic training for enhancing game-specific skill acquisition and perceptual-cognitive agility, they should be integrated with SAQ or HIIT to address deficits in maximal linear speed and locomotor overload that SSGs alone may not provide [1, 9, 23, 39]. Furthermore, practitioners can employ differential learning or pre-session fatigue protocols to challenge technical execution and decision-making under stress, fostering greater tactical adaptability [4]. For optimal technical outcomes, SSGs should be used as the primary methodological resource, supplemented by isolated drills or combined protocols (e.g., SSG + core strength) only when specific technical volume or explosive power cannot be consistently achieved through gameplay [1, 20, 28].

Rule manipulations have also been reported to influence technical interaction density. Touch limitations (e.g., 1- or 2-touch rules) have been associated with increased ball circulation and higher player interaction frequency [2, 6, 31]. However, stricter touch restrictions, particularly 1-touch rules, have also been associated with higher technical error rates and lower execution accuracy in youth or less experienced players [2, 28, 31]. Defensive instructions such as man-to-man marking have also been reported to increase technical pressure on the ball carrier compared with zonal defensive structures [6, 17].

Directional structure and scoring rules may also influence the distribution of technical actions. Single-goal formats have been reported to increase shooting frequency and defensive coverage, although some studies report higher passing error rates in these conditions [16]. The use of multiple mini-goals has been associated with greater use of lateral spaces and higher frequencies of dribbling and switching actions during play [16, 19]. Similarly, “Score Zone” (stop-ball) formats have been reported to increase attacking involvement and individual participation compared with traditional goal formats [6, 16].

Work-bout duration has also been reported to influence technical activity during SSGs. Several studies describe intermittent bouts lasting approximately 2.5–4 minutes during SSG-based training tasks [3, 6, 23, 24]. These formats have been associated with repeated technical involvement while limiting reductions in execution accuracy that may occur during prolonged high-intensity play [20, 28]. Several reviews also report that SSG-based training provides frequent opportunities to perform sport-specific technical actions in representative game environments [1, 13]. Some studies also describe the use of complementary learning approaches, such as differential learning tasks, alongside SSG training to increase variability in technical and tactical responses [4, 28].

### Tactical Behaviour Outcomes

As summarized in Table 7, several reviews report that collective tactical behaviors in SSGs vary according to pitch dimensions, player format, and directional task constraints [10, 17, 36]. Larger pitch dimensions and greater area per player have been associated with increases in team dispersion metrics such as team length, width, surface area, and stretch index [8, 10, 17, 19]. In contrast, smaller formats (e.g., 1 v 1–4 v 4) and reduced playing spaces have been associated with higher levels of team compactness, shorter interaction sequences, and increased local interactions between players or sub-units [4, 8, 9, 12, 22].

**TABLE 7:** Evidence-Based Design Blueprint for Small-Sided Games Targeting Tactical Behaviors / Game Principles.

Design components	Tactical Behaviors / Game Principles (compactness, spacing, pressing behaviors, transitions)
**Area per Player** (playing space available per player; pitch size manipulation)	ApP is a fundamental constraint that dictates the spatial scale and collective organization of the game [10, 19]. Synthesized evidence suggests that smaller pitches are ideal for tactical exploration in youth or low-skill groups as they reduce task complexity [8]. Furthermore, smaller spaces promote higher irregularity in spatial distribution, which is associated with greater tactical adaptability [12]. In contrast, larger pitch dimensions significantly increase team spread, interpersonal distances, and effective playing space, as evidenced by higher Stretch Index and Surface Area values [8, 19]. These larger areas are necessary to foster collective tactical behaviors, such as attacking/defensive unity and spatial organization, while smaller areas enhance individual tactical skills, such as penetration and defensive delays [4, 22]. Therefore, practitioners should align the spatial scale with the desired tactical outcome: match-representative dimensions for collective unity and micro-structures (e.g., 1 v 1) for individual tactical development [4].

**Format / Number of Players** (team size configuration; e.g., 2 v 2, 3 v 3, 4 v 4, 6 v 6; player density)	Format and team size are decisive factors in determining whether tactical training focuses on individual skills or collective organization [19, 22]. Synthesized evidence indicates that smaller formats (e.g., 1 v 1 to 3 v 3) are superior for developing individual tactical behaviors, specifically penetration, individual duels, and defensive delays [19, 22]. These micro-structures significantly increase the frequency of technical-tactical transitions and exploratory behaviors compared to larger games [2, 5]. Conversely, larger formats (e.g., 5 v 5 to 10 v 10) are more effective for fostering collective tactical principles, such as attacking and defensive unity, team compactness, and positional regularity [2, 22]. While the 3 v 3 format is highlighted as an optimal balance for technical-tactical integration and ball possession [18, 28], 4 v 4 and 5 v 5 formats are specifically recommended for training tactical endurance and stabilizing locomotor demands [20].

**Bout Duration** (length of repetitions or continuous play intervals)	Bout duration is a critical factor in ensuring that the quality of tactical decision-making and collective organization does not degrade due to physical or mental fatigue [6, 20, 26]. Synthesized evidence indicates that tactical-focused SSGs typically use intervals of 2.5 to 4 minutes per set to maintain high intensity and precision [23, 24]. Implementing intermittent protocols (e.g., 4 sets of 4 minutes) is superior to continuous play for preserving the accuracy of collective tactical actions, such as offensive unity and defensive synchronization [3, 17]. For micro-formats (1 v 1 to 3 v 3) that target rapid tactical reactions and responsiveness, practitioners should use shorter, high-intensity bouts [20]. Furthermore, coaches should be mindful that mental fatigue induced prior to training can specifically reduce the accuracy of offensive tactical behaviors, making shorter and highly focused bouts more effective during periods of high cognitive load [26].

**Work-to-Rest Ratio** (ratio between active playing time and recovery time; interval structure)	The work-to-rest ratio is a critical determinant of the precision and cognitive quality of tactical decision-making by managing physical and mental load [17, 20]. Evidence suggests that utilizing intermittent structures, such as 4 sets of 4-minute games with 3-minute recovery periods, is effective for sustaining tactical engagement and accuracy across the session [3]. Implementing multiple active breaks within longer game protocols further enhances overall work capacity, ensuring that collective dynamics remain stable under metabolic strain [34]. For high-intensity micro-formats (e.g., 1 v 1 to 3 v 3), longer rest intervals are particularly vital to mitigate catabolic stress and prevent the performance decay that leads to tactical errors and reduced responsiveness [20].

**Rules / Constraints** (pressing constraints, scoring/target constraints, zonal constraints, conditioning constraints)	Rules and structural constraints are strategic manipulations used to modulate tactical intensity, collective organization, and the frequency of individual interactions [17, 19, 36]. Implementing man-to-man marking significantly increases tactical demands and internal/external load compared to zonal defensive structures [6, 17]. Scoring targets serve as critical constraints; single-goal formats enhance defensive grouping, whereas multiple-goal configurations (e.g., 2 or 4 mini-goals) promote lateral movement, offensive variety, and the exploitation of defensive width [16, 19]. The “Score Zone” (stop-ball) rule is an effective modification that increases attacking volume and metabolic strain compared with traditional goals [6, 16]. Numerical imbalances (e.g., 4 v 3) are highly effective for fostering exploratory behavior and tactical variability, with attacking teams in superiority expanding the playing space while defensive teams in inferiority focus on compactness and goal protection [2, 5, 12, 19]. Furthermore, touch limitations (e.g., one or two touches) amplify technical-tactical interaction density and the urgency of support play, although they may increase error rates in developing players [2, 6, 31]. Finally, coach instructions and verbal encouragement can further modulate team dispersion, compactness, and the emergence of specific tactical principles [6, 22, 28].

**Touch Limitation** (one-touch, two-touch, limited touches, free touches)	Touch limitations (e.g., one-touch or two-touch) are powerful constraints for amplifying the speed of tactical decision-making and the density of technical-tactical interactions [2, 31]. These restrictions specifically force players to improve their off-the-ball movement and create passing lanes more aggressively at higher intensities to maintain ball circulation [6, 31]. While effective for increasing the urgency of support play, practitioners should be aware that strict touch limits often lead to higher technical error rates, which can disrupt collective tactical regularity and possession stability, particularly in youth or less experienced populations [2, 28, 31]. Therefore, touch constraints should be periodized to balance the need for high-speed decision-making with the maintenance of tactical organization [2].

**Joker Usage** (inside jokers, outside jokers, neutral players; numerical superiority)	Joker usage and numerical imbalances (e.g., 4 v 4 + 2 floaters) are strategic manipulations that significantly alter tactical density and the density of technical-tactical interactions [2, 12]. Teams in numerical superiority (on the attacking side) tend to expand the playing area in both width and depth, which facilitates positional regularity and successful ball retention [2, 19, 22]. Conversely, playing in numerical inferiority (on the defensive side) promotes greater exploratory behavior and tactical variability, forcing players to adapt their technical-tactical solutions under disadvantageous conditions [5, 12]. It is important to note that adding neutral players to a fixed pitch size reduces the relative ApP, potentially increasing tactical congestion; therefore, pitch dimensions should be adjusted proportionally to maintain the desired tactical load [10]. Finally, numerical imbalances are highly effective for teaching specific sub-phase goals, such as defensive coverage during inferiority or penetration during superiority [19, 22].

**Directionality & Goalkeeper Involvement** (directional play, goal-oriented tasks, presence or absence of the goalkeeper)	Directionality and goalkeeper involvement are key determinants of tactical focus, as they define the spatial orientation and collective purpose of the training task [16, 19]. Single-goal formats are particularly effective for fostering defensive organization and grouping [16]. In contrast, utilizing multiple targets (e.g., 2 or 4 mini-goals) shifts the play toward lateral corridors, encouraging players to exploit defensive width and increase offensive variety [16, 19]. The implementation of a “Score Zone” (stop-ball) rule is effective for increasing attacking volume and metabolic strain compared to traditional goals [6, 16]. Furthermore, manipulating goal positioning, such as diagonal or atypical placements, serves as a potent constraint that provokes original tactical behaviors and creative movements [5]. While goalkeepers enhance tactical realism, their inclusion reduces the relative ApP, potentially increasing tactical congestion unless pitch dimensions are adjusted [10, 19].

**Top-Up Methods** (applied conditioning add-on strategies)	Tactical adaptability and creativity are significantly enhanced through the application of differential learning and non-linear pedagogy, which foster improvements across all tactical dimensions [4]. Coaches should utilize specific offensive and defensive instructions to effectively modulate team dispersion, compactness, and the emergence of collective principles [22]. However, practitioners should be mindful that coach presence and verbal pressure may increase player stress, potentially leading to a decrease in the percentage of successful passes [28]. To provoke original tactical solutions and exploratory behavior, practitioners can implement “constraints-led” strategies such as atypical goal positioning (e.g., diagonal goals) or numerical imbalances [5, 12]. While mental fatigue induced prior to training does not acutely impair running volume, it can reduce the accuracy of collective behaviors, such as offensive unity; therefore, fatigue-inducing tasks can be strategically used to challenge decision-making under stress [4, 26]. Finally, since SSGs often provide inconsistent stimuli for maximal speed, they should be supplemented with HIIT or isolated sprint drills to ensure that physical limitations do not hinder the execution of tactical organization in larger spaces [9, 17, 24, 27, 39].

Player format has also been reported to influence the scale of tactical interactions. Micro-formats (1 v 1 to 3 v 3) have been associated with higher frequencies of individual tactical actions such as 1 v 1 duels, penetration attempts, and defensive delays [4, 19, 22]. Larger formats (5 v 5 to 10 v 10) have been associated with greater expression of collective tactical behaviors, including coordinated attacking movements, defensive organization, and positional spacing [2, 22]. Several studies also report that numerical imbalances (e.g., 3 v 4 or 4 v 5) are associated with increased variability in tactical behaviors and greater exploratory movement patterns during play [2, 5, 12].

Directional structure and rule manipulations may also influence tactical organization. Single-goal formats have been reported to increase defensive grouping and central defensive coverage. In contrast, the use of multiple mini-goals has been associated with greater lateral ball circulation and wider spatial use during attacking phases [16, 19]. Task constraints such as atypical goal positioning (e.g., diagonal goal placement) have also been reported to alter players’ spatial positioning and movement patterns during tactical interactions [5]. Touch restrictions and man-to-man marking have been associated with increases in off-the-ball movement and passing interactions compared with free-play conditions [2, 6, 31].

Work-bout duration has also been described in relation to tactical activity during SSGs. Several studies report the use of intermittent bouts lasting approximately 2.5–4 minutes during SSG training tasks [23, 24]. Shorter intermittent formats have been associated with the maintenance of tactical activity during play. In contrast, prolonged continuous play may be accompanied by reductions in the accuracy of coordinated offensive and defensive actions under fatigue conditions [3, 17, 26].

### Summary of Key Design Components

As summarized in Table 8, several reviews report associations between key SSG design components and performance outcomes across different training domains. Area per player has frequently been reported in relation to changes in high-speed running (HSR), sprint exposure, and team dispersion metrics [8, 10, 17]. Increasing area per player (typically > 200 m^2^ per player) has been associated with higher high-velocity running distances and greater team spatial dispersion (e.g., stretch index and surface area), alongside moderate increases in mean heart rate responses [8, 10, 27]. In contrast, smaller pitch areas (< 100 m^2^ per player) have been associated with higher frequencies of accelerations, decelerations, and repeated technical actions [8, 18, 36].

**TABLE 8:** Major, Secondary, and Negligible Roles of Each SSG Design Component Across Six Performance Objectives.

Design components	Performance Objectives
**Area per Player** (playing space available per player; pitch size manipulation)	ApP is a fundamental constraint in small-sided games (SSG) design, serving as a major determinant across most performance objectives. For Aerobic and Internal Load, ApP plays a major role, as larger relative areas (typically > 100 m^2^/player) significantly increase mean heart rate, RPE, and blood lactate levels [6, 8, 10]. Similarly, it is a major factor for External Load (HSR and Sprinting); increasing the pitch size leads to higher high-speed running distances and total distance, though match-representative intensities for high-speed running and sprinting often require areas exceeding 200 m^2^/player and 365 m^2^/player, respectively [8, 10, 27]. Regarding Neuromuscular Load (ACC/DEC), ApP is also major because reducing the playing space is a primary strategy to maximize the density of acceleration and deceleration actions [8, 36]. For Technical Execution, ApP plays a major role, with smaller pitch areas essential for increasing the frequency and variability of individual actions, such as passes, dribbles, and ball control [2, 19, 28]. In terms of Tactical Behaviors, larger pitches are considered major, as they increase team spread (Stretch Index) and collective unity, while smaller areas are vital for fostering tactical exploration and adaptability [8, 12, 19, 22]. In contrast, ApP plays a secondary role in developing Agility and COD; while smaller spaces increase movement frequency, these outcomes are often more strongly influenced by the number of players and specific rules than by spatial manipulation alone [8, 9, 35]. Finally, the impact of ApP on maximal linear speed and vertical power (jumping) is generally negligible, as SSGs typically provide insufficient longitudinal distance and lack the standardized acceleration sequences required for these specific neuromuscular adaptations [9, 17, 24, 27, 39].

**Format / Number of Players** (team size configuration; e.g., 2 v 2, 3 v 3, 4 v 4, 6 v 6; player density)	Format / Number of Players is a primary design component that dictates the balance between individual technical engagement and collective tactical organization. For Aerobic and Internal Load, team size plays a major role, with smaller formats (1 v 1 to 4 v 4) eliciting significantly higher physiological strain (85–90% HRmax) and blood lactate levels compared to larger formats [6, 18, 20]. In terms of Technical Execution, it is also a major determinant; reducing the number of players (e.g., from 4 v 4 to 2 v 2) increases individual ball engagement and the frequency of technical actions such as passes, dribbles, and shots [2, 19, 28, 29]. For Tactical Behaviors, format size has a major impact on the scale of play: micro-formats (1 v 1 to 3 v 3) are essential for individual tactical skills like penetration and defensive delays, while larger formats (5 v 5 to 10 v 10) are required for training collective unity, team compactness, and positional regularity [19, 22]. Additionally, player numbers play a major role in Neuromuscular Load (ACC/DEC) and Agility/COD, as smaller formats increase the density of accelerations, decelerations, and rapid directional changes [9, 36, 38]. Conversely, format size plays a secondary role for External Load (HSR and Sprinting); while 4 v 4 to 6 v 6 formats generally cover more total distance and high-speed running than 2 v 2 due to greater longitudinal space, these distances remain significantly lower than those found in 11-a-side matches [10, 18, 27]. Finally, the effect of player numbers on maximal linear speed is negligible, as even larger formats do not consistently reach the maximal sprint thresholds needed for linear speed adaptation [9, 24, 39].

**Bout Duration** (length of repetitions or continuous play intervals)	Bout Duration (the length of each individual set) plays a major role in managing Aerobic and Internal Load; synthesized evidence suggests that utilizing intermittent structures, such as 4 sets of 4-minute games, is a primary strategy for maintaining high physiological strain (85–90% HRmax) and achieving aerobic adaptations comparable to conventional endurance training [3, 6, 23]. It is also a major determinant for Tactical Behaviors, as maintaining set durations between 2.5 and 4 minutes is critical for preserving the quality of decision-making and collective organization; durations exceeding these thresholds may induce physical and mental fatigue that specifically reduces the accuracy of offensive unity and defensive synchronization [6, 24, 26]. Regarding External Load, bout duration plays a secondary role, with intermittent regimens generally superior to continuous play for accumulating higher volumes of high-intensity running distance while providing necessary recovery to maintain movement quality [17, 20]. Similarly, it plays a secondary role in Technical Execution by ensuring that technical actions, such as passing, shooting, and ball control, are performed with high precision and variability before performance decays due to fatigue [28, 29]. In contrast, its impact on maximal linear speed and neuromuscular power (jumping) is negligible, as manipulating the duration of a set does not address the lack of longitudinal space and standardized acceleration sequences required for these specific adaptations [24, 39].

**Work-to-Rest Ratio** (ratio between active playing time and recovery time; interval structure)	Work-to-Rest Ratio is a critical design component that functions as a major determinant for Aerobic and Internal Load; evidence indicates that implementing intermittent protocols (e.g., 4 minutes of work with 3 minutes of rest) is a primary strategy for maintaining high-intensity physiological strain (85–90% HRmax) throughout a session [3, 17, 23]. It also plays a major role in Tactical Behaviors, as sufficient recovery intervals are necessary to mitigate the onset of physical and mental fatigue, thereby preserving the quality of decision-making and collective organization [17, 20]. In high-intensity micro-formats like 1 v 1, longer rest intervals are particularly vital to prevent performance decay and reduce the risk of overtraining [20]. Regarding Technical Execution, the work-to-rest ratio has a secondary role, primarily by ensuring that fatigue does not lead to high technical error rates, although the absolute volume of technical actions is more heavily influenced by format and pitch size [2, 28, 31]. Similarly, it serves a secondary role in External Load (HSR and Sprinting); while intermittent regimens allow for greater accumulation of high-speed running distance than continuous play, they cannot fully compensate for the lack of longitudinal space inherent in small formats [17]. Finally, its impact on maximal linear speed and neuromuscular power is negligible, as rest periods do not address the lack of standardized acceleration sequences and distance required for maximal velocity adaptations [1, 16].

**Rules / Constraints** (pressing constraints, scoring/target constraints, zonal constraints, conditioning constraints)	Rules and Constraints (comprising pressing styles, scoring/target methods, zonal restrictions, and conditioning instructions) serve as a major determinant for Tactical Behaviors, as they are the primary strategic tools used to modulate collective organization and the emergence of specific game principles [17, 19, 22]. Scoring targets are a major factor in defining spatial orientation; while single-goal (1G) formats favor defensive grouping and organization, the use of multiple targets (e.g., mini-goals) shifts play toward lateral corridors and encourages players to exploit defensive width [16, 19]. Furthermore, atypical constraints, such as diagonal goal positioning, are major drivers for provoking original tactical solutions and creative exploratory movements [5]. These constraints also play a major role in Aerobic and Internal Load. Implementing man-to-man marking significantly increases physiological demands compared to zonal systems [6, 17]. Scoring modifications are equally impactful; the “Score Zone” (stop-ball) rule elicits significantly higher heart rate (~90% HRmax) and metabolic strain than traditional goal formats [16]. Additionally, external conditioning factors like coach presence and verbal encouragement are major facilitators for amplifying session intensity and maintaining high levels of internal load [6, 28]. Regarding Technical Execution, rules and constraints play a secondary role by influencing the distribution and variety of technical actions; for instance, 1G formats tend to increase inaccurate passes but promote specific skills like shooting and defensive coverage [2, 16, 28]. They also have a secondary impact on External Load (Total Distance and HSR) and Agility/COD. While specific rules like man-to-man marking increase the volume of high-intensity movement, the overall locomotor load remains fundamentally governed by the pitch dimensions and number of players [6, 10, 16, 17]. Finally, the impact of rules and constraints on maximal linear speed and neuromuscular power is negligible, as no rule manipulation can compensate for the lack of longitudinal space and standardized acceleration paths required for maximal velocity development [24, 27, 39].

**Touch Limitation** (one-touch, two-touch, limited touches, free touches)	Touch Limitation is a high-impact design constraint that is a major determinant of Aerobic and Internal Load; research indicates that restricting touches (e.g., 1-touch or 2-touch) significantly increases physiological strain, resulting in higher blood lactate levels and elevated RPE across various formats [6, 31]. It also plays a major role in Technical Execution, as it forces a higher density of technical-tactical interactions and increases the speed of ball circulation, though practitioners should note it often leads to higher technical error rates [2, 28, 31]. In terms of Tactical Behaviors, touch limits are considered major because they demand superior off-the-ball movement and more aggressive creation of passing lanes, which accelerates collective decision-making and transitions [2, 6, 31]. Conversely, touch limitations generally play a secondary role in External Load (Total Distance and HSR); most synthesized evidence indicates that these restrictions do not significantly alter total distance covered compared with free-play, as locomotor load is more heavily determined by pitch dimensions [6, 31]. They also have a secondary impact on Agility and Neuromuscular responsiveness by increasing the frequency of short, sharp movements and rapid reactions required to maintain possession under pressure [6, 20]. Finally, the impact of touch limits on maximal linear speed and vertical power is negligible, as these constraints do not overcome the lack of longitudinal space or the specific mechanics required for maximal velocity adaptations [24, 39].

**Joker Usage** (inside jokers, outside jokers, neutral players; numerical superiority)	Joker Usage and Numerical Imbalance (e.g., 4 v 4 + 2 floaters) are major determinants of Tactical Behaviors, as these manipulations are primary tools for provoking specific game principles and exploratory actions [2, 5, 12, 19]. Research indicates that teams in numerical superiority (attacking side) tend to expand the playing area in both width and depth, facilitating positional regularity and ball retention, while teams in numerical inferiority (defensive side) focus on compactness and goal protection [2, 19, 22]. Furthermore, playing with a numerical disadvantage significantly increases exploratory behavior and tactical variability, forcing players to find creative technical-tactical solutions under pressure [5, 12]. This component also plays a major role in Technical Execution, as the presence of “free” players (jokers) increases the density of successful passes and technical-tactical transitions by providing constant support for the team in possession [2, 19]. In contrast, Joker Usage plays a secondary role in both Aerobic /Internal Load and External Load. While adding floaters alters the intensity of the game, its impact is often tied to how it changes the relative ApP; adding players to a fixed pitch reduces the available space per player, which may increase tactical congestion and inadvertently decrease high-speed running unless pitch dimensions are adjusted proportionally [10]. Similarly, it has a secondary impact on Agility and Neuromuscular responsiveness, as the increased interaction density requires more frequent short-distance adjustments [6]. Finally, its impact on maximal linear speed and neuromuscular power is negligible, as numerical manipulations do not provide the necessary longitudinal distance or standardized sprint mechanics required for maximal velocity development [24, 27, 39].

**Directionality & Goalkeeper Involvement** (directional play, goal-oriented tasks, presence or absence of the goalkeeper)	Directionality and Goalkeeper Involvement (including scoring methods such as mini-goals, stop-ball, or traditional goals) are major determinants of Tactical Behaviors, as they define the spatial orientation and collective purpose of the game [16, 19]. Utilizing multiple targets (e.g., 2 or 4 mini-goals) is a primary strategy for shifting play toward lateral corridors and encouraging players to exploit defensive width, whereas single-goal formats are superior for fostering defensive organization and grouping [16, 19]. Strategic goal positioning (e.g., diagonal goals) is also a major factor in provoking original tactical solutions and creative movements [5]. Additionally, the inclusion of goalkeepers enhances tactical realism but reduces the relative ApP, potentially increasing tactical congestion unless pitch dimensions are adjusted [10, 19, 22]. This component also plays a major role in Aerobic and Internal Load; specifically, stop-ball (Score Zone) formats elicit significantly higher physiological strain (~90% HRmax) and RPE compared to traditional goals, while 1-goal games generally induce higher internal load than multiple-goal configurations [6, 16]. Regarding Technical Execution, directionality plays a secondary role, influencing the variety and accuracy of technical actions; for instance, 1-goal formats may increase inaccurate passes but favor specific sub-phase skills, such as shooting or defensive coverage [16]. It also has a secondary impact on External Load (Total Distance and HSR) and Agility/COD; while the scoring objective and target density can modulate movement intensity and reaction frequency, the overall locomotor load is more fundamentally governed by the pitch dimensions and number of players [6, 10, 16, 20]. Finally, its impact on maximal linear speed and neuromuscular power is negligible, as changing the scoring method or adding goalkeepers does not address the lack of longitudinal distance and standardized acceleration sequences required for maximal velocity development [24, 27, 39].

Player format (number of players) has also been reported to interact with spatial constraints in shaping locomotor and tactical responses. Larger formats (≥ 6 v 6) have been associated with longer running corridors, greater exposure to high-speed running, and greater expression of collective tactical behaviors [18, 22, 29]. Micro-formats (1 v 1 to 3 v 3) have been associated with higher frequencies of individual duels, rapid technical–tactical interactions, and short explosive movements [2, 18–20, 22]. Some reviews also report that formats such as 4 v 4 are frequently used in training interventions combining substantial cardiovascular load with moderate locomotor demands [18, 29].

Work-bout duration and work-to-rest (W:R) ratios have also been described in relation to metabolic responses during SSG training. Several studies report the use of intermittent bouts lasting approximately 4 minutes during protocols targeting aerobic responses, often resulting in heart rate values of ~85–90% HRmax [3, 6, 17, 23]. Shorter bouts lasting approximately 30–90 seconds combined with short recovery intervals (e.g., 1:1–1:2 ratios) have been reported in protocols targeting glycolytic or repeated high-intensity actions [13, 21, 38].

Rule manipulations and task constraints have also been reported to influence internal load and movement patterns during SSGs. Touch restrictions (e.g., 1- or 2-touch rules) and man-to-man marking have been associated with increases in internal load responses and movement intensity during play [6, 17, 31]. Directional structures, including the presence of goals or goalkeepers, have also been reported to influence spatial organization and forward progression during SSG tasks [16, 17, 19]. Several reviews also report that SSG formats may provide limited exposure to maximal sprint speeds compared with match play [9, 15, 24, 27, 39].

## DISCUSSION

This umbrella synthesis contributes to the soccer literature by translating 37 systematic reviews and meta-analyses into an evidenceinformed blueprint [1–10, 12, 13, 15–39]. By aggregating findings from review-level evidence rather than individual primary studies, the present work provides a higher-order synthesis of the current SSG literature. It clarifies how commonly manipulated task constraints relate to specific performance outcomes. This framework links eight core design levers—relative area per player, player format, bout duration, work-to-rest ratio, rules and task constraints, touch limitations, joker usage, and directionality—with predefined training objectives, organizing these links into a practitioner-oriented decision matrix [13, 25, 36]. Rather than re-estimating effects from primary studies, this review integrates convergent and recurring patterns across six primary performance domains: aerobic load, anaerobic/RSA, HSR/sprint, ACC–DEC/CoD, technical execution, and tactical behaviors [9, 12, 23, 33]. In this sense, the synthesis does not attempt to replace existing quantitative meta-analyses but instead contextualizes their collective findings within a practical training-design perspective. Where appropriate, the framework offers theory-informed interpretations of how spatial and temporal constraints function as primary regulators of internal and external load. In contrast, rule-based and informational constraints serve as secondary modifiers of technical and tactical emergence [2, 29].

In doing so, the synthesis aims to narrow the gap between quantitative evidence summaries and practical training decisions, while acknowledging that SSG-only exposures often provide an insufficient stimulus for maximal linear speed and sprint density compared to official match demands [17, 25, 27]. This observation is consistent with the results reported in the present synthesis, which showed that high-speed running and maximal sprint exposures during SSG formats remain substantially lower than those observed during full-sided competitive matches. The blueprint also highlights persistent methodological limitations in the literature, including inconsistent reporting of speed-threshold definitions, variation in area-per-player calculation conventions (e.g., including or excluding goalkeepers), and incomplete disclosure of work-to-rest ratios, all of which constrain cross-study comparability [7, 27]. Such methodological variability likely contributes to the wide betweenstudy variability observed across several SSG outcomes, particularly for locomotor metrics such as HSR and sprint exposure. Accordingly, the decision matrix is presented as a transparent and updateable framework, with recommendations explicitly interpreted in light of evidence heterogeneity and the under-representation of female, youth, and elite professional cohorts [32, 38]. Rather than a definitive prescriptive tool, the matrix serves as a flexible logic to support context-sensitive session design and the strategic integration of supplementary “top-up” conditioning [27, 29, 39].

### Interpretation of Aerobic Load

Across small- to large-sided game formats (SSG–MSG–LSG), this synthesis of 37 systematic reviews and meta-analyses suggests that SSG-based training provides a high, programmable aerobic stimulus, frequently eliciting internal load responses comparable to those of running-based HIIT [18, 22, 26]. Meta-analytic evidence commonly reports average session intensities of ~78–84% HRmax, with approximately 12–30% of session time spent above the 90% HRmax threshold [4]. Chronic adaptations across 3–12-week interventions yield pooled ${\dot{\text{V}}\text{O}}_{2\max}$ gains of approximately +3.5 mL · kg^−1^ · min^−1^, indicating that SSGs represent a viable, time-efficient alternative to HIIT for aerobic development when session density and duration are aligned [4, 34]. These observations are consistent with the results of the present synthesis, which showed that most SSG formats can elicit internal loads within commonly accepted aerobic training zones.

From a programming perspective, the interaction between spatial and temporal constraints serves as the primary regulator of aerobic strain. Previous syntheses have consistently highlighted the importance of pitch size and player density in shaping cardiovascular responses during SSGs, and the present findings reinforce this relationship [8, 17]. To maximize cardiovascular adaptations, an area per player of 100–150 m^2^ is recommended; staying above the 100 m^2^ threshold is critical for sustaining the continuous physiological strain necessary for aerobic flow [39]. Smaller formats (2 v 2 to 4 v 4) are identified as the most effective for consistently reaching 85–90% HRmax, with the 4 v 4 format highlighted as a “gold standard” for balancing high internal load with match-representative running volumes [18]. Regarding temporal structure, 4-minute work bouts are considered the gold standard for developing aerobic power [3, 22]. Intermittent regimens utilizing 2–3-minute recovery periods (approximate ratios of 1:1 to 4:3) are superior to continuous play for maintaining heart rates within target zones while facilitating higher external load accumulation [3, 17, 22].

From a physiological standpoint, the aerobic loads reported are consistent with established mechanisms of adaptation, such as improvements in stroke volume, capillarization, and mitochondrial enzyme activity [22, 31]. Repeated exposure to these intensities is known to stimulate central and peripheral adaptations that collectively enhance oxygen delivery and utilization during exercise, which helps explain the aerobic improvements observed across SSG interventions. These mechanisms are triggered when athletes repeatedly experience high-intensity aerobic strain with incomplete recovery. From an ecological-task perspective, rule-based constraints further modulate intensity: man-to-man marking, coach encouragement, and “Score Zone” (stop-ball) rules significantly amplify metabolic urgency, with heart rate responses often reaching ~90% HRmax [6, 8, 16, 19].

For practitioners, these findings support the placement of aerobic-oriented SSGs on MD–4 or MD–3 within the weekly microcycle to maximize physical acquisition [7, 18]. Monitoring should include HR-derived metrics alongside RPE to verify the intended stimulus, especially since joker usage can inadvertently reduce physiological strain if pitch dimensions are not increased proportionally [10, 18, 19]. Finally, it must be acknowledged that while SSGs are highly effective for aerobic goals, they often provide insufficient exposure to maximal linear sprinting, necessitating the strategic integration of “topup” conditioning to ensure match-representative locomotor overload [15, 17, 24, 25, 27]. This limitation reflects one of the recurring findings of the present synthesis, particularly within the HSR and sprint domains, where SSG exposures consistently remained below competitive match demands.

### Interpretation of Anaerobic/Glycolytic and RSA Outcomes

This synthesis suggests that SSG-based training, when structured through small formats (1 v 1 to 3 v 3) and reduced relative area per player (approximately 50–100 m^2^ · player^−1^), elicits a pronounced anaerobic–glycolytic stimulus comparable to running-based HIIT [6, 18, 20, 27]. Meta-analytic data in team-sport cohorts report that 4–12-week SSG interventions yield large improvements in RSA total time (SMD ≈ −1.19) and moderate gains in 10–20 m sprint performance (SMD ≈ −0.89), with non-significant differences compared to HIIT protocols [27]. These findings align with the results of the current synthesis, which show that micro-format SSG designs consistently generate high densities of accelerations, decelerations, and multidirectional actions. Time–space compression achieved through micro-formats and task constraints (e.g., pressing or 1-touch rules) is associated with significantly higher blood lactate concentrations (> 6 mmol/L) and increased ACC–DEC and change-of-direction density [6, 18, 27].

From a programming standpoint, the evidence indicates that short work bouts (30 seconds to 2 minutes) with brief recoveries (1:1 to 1:2 ratios) are the most effective SSG configurations for targeting maximal anaerobic thresholds and glycolytic stress [13, 20, 35]. These intermittent structures allow players to maintain a high density of explosive mechanical actions and sport-specific responsiveness without the intensity drift associated with longer work durations [20, 24]. This observation reflects the time–motion characteristics of match play, where repeated high-intensity efforts are typically interspersed with short recovery periods rather than sustained continuous activity. However, the synthesis highlights that anaerobic-focused SSGs provide highly variable exposure to high-speed running, necessitating the use of standardized speed thresholds and explicit reporting of work-to-rest ratios to ensure training quality [7, 27].

From a mechanistic perspective, plausible adaptive mechanisms triggered by these formats include greater high-threshold motor-unit recruitment, elevated glycolytic flux, and improved buffering capacity under repeated high-intensity efforts [6, 41]. Repeated exposure to these high-intensity, intermittent demands may also improve phosphocreatine resynthesis efficiency and enhance tolerance to metabolic acidosis during repeated sprint sequences. For practitioners, when the primary aim is to maximize anaerobic density, these sessions should be placed on acquisition days (MD–3 or MD–4) and may be complemented with brief post-SSG “top-ups” (e.g., 15–30 s linear sprints or RSA blocks) to secure match-representative sprint density [7, 27, 29, 38]. Session monitoring should ideally combine external mechanical metrics (ACC–DEC or CoD counts) with internal indicators such as RPE and time spent above 90% HRmax to confirm the intended metabolic stimulus [13, 20, 21].

### Interpretation of High-Speed Running and Sprint Exposure

This synthesis identifies relative area per player and format size as the most influential design levers for modulating HSR and sprint exposure in SSGs [10, 17, 36]. A clear dose-response relationship exists where every 25 m^2^ increase in area per player correlates with a 1.9–2.8 m/min rise in HSR exposure across all formats [27]. This relationship aligns with the findings summarized in the Results section, where larger pitch dimensions consistently produced greater high-speed locomotor outputs across SSG formats. To replicate matchrepresentative HSR intensities, pitch dimensions must exceed 200 m^2^/player, while reaching maximal sprinting thresholds (sprints > 25.1–25.2 km/h) typically requires an area per player exceeding 300–365 m^2^/player with 35–40 m longitudinal corridors [8, 10, 27, 38]. Mid-to-large formats (e.g., 6 v 6 to 9 v 9) are generally superior to micro-formats for these objectives, as they provide the uninterrupted running lanes and longer acceleration distances necessary to elicit high-velocity actions [6, 18, 39].

Meta-analytic profiling reports typical HSR values of 14.8–17.2 m · min^−1^, very high-speed running (VHSR) of ≈2.7–3.6 m · min^−1^, and sprint exposure of ≈0.2–0.7 m · min^−1^ [7, 27]. These outputs are characterized by substantial between-session variability (CV ≈ 22.8%) and are highly sensitive to velocity-threshold definitions and task orientations [7, 21, 27]. Such variability likely reflects differences in pitch geometry, player density, and tactical constraints, which have been repeatedly identified as key moderators of locomotor output in SSG research. Accordingly, convergent reviews emphasize the need to standardize speed thresholds and explicitly report work-to-rest ratios—ideally using 1:3 to 1:6 ratios for locomotor targets—to ensure training quality and preserve peak velocities [17, 20, 21, 27].

From an applied perspective, multiple syntheses confirm that HSR and sprinting exposure across all common-sided game formats remain consistently significantly lower than the demands of competitive 11-a-side matches [17, 27]. Consequently, SSGs alone are often insufficient for maximal linear speed development and hamstring injury prevention [25, 27]. This discrepancy between training and match exposure has been widely discussed in the locomotor load literature, where insufficient exposure to maximal sprinting has been linked to increased hamstring injury risk and reduced sprint preparedness. For practitioners, brief post-SSG “topups,” such as linear sprint repetitions, RSA blocks, or HIIT, are essential strategies to secure peak sprint density while preserving sport-specificity [15, 24, 27, 29, 38].

Rule manipulations and structural constraints can further refine the locomotor profile. Directional play with goalkeepers, man-to-man marking, and “Score Zone” (stop-ball) rules foster higher movement urgency and vertical transitions, helping to maximize the available HSR stimulus within the drill [6, 16, 19]. Within the weekly microcycle, high-intensity HSR and sprint exposure should peak on MD– 3, which serves as the primary acquisition day for explosive external load [7]. Such periodization strategies are consistent with contemporary match-load management models, which aim to align training stimuli with the physical demands of competition. Collectively, these findings highlight that while spatial upscaling is the primary driver for high-velocity running, the strategic integration of supplementary conditioning is required to bridge the gap between SSG-induced loads and match-play demands [17, 25, 27, 39].

### Interpretation of Acceleration–Deceleration and CoD Mechanisms

This synthesis suggests that small formats (1 v 1–3 v 3) combined with reduced relative area per player (approximately ≤ 75–100 m^2^ · player^−1^) and time–space–compressing task constraints are the primary drivers of acceleration–deceleration density and CoD demands [17, 20, 36, 38]. This pattern is consistent with the results reported in the present synthesis, where compact pitch configurations produced the highest densities of accelerations, decelerations, and multidirectional movements. Unlike high-speed running, which requires expansive pitch dimensions, increasing the playing area (e.g., > 250 m^2^/player) does not significantly amplify the volume of explosive mechanical bursts [8, 10]. Consequently, compact fields and micro-formats are superior for promoting the high-intensity footwork and reactive bursts critical for game-specific agility [17, 20, 36, 38].

Meta-analytic evidence indicates that SSG-based interventions are highly effective at enhancing multidirectional speed, resulting in significant improvements in CoD performance over 4–8 weeks [33–35, 37]. These effects are often of similar magnitude to those observed in running-based HIIT or RSA blocks [33, 37, 38]. This finding supports the idea that repeated exposure to high densities of acceleration, braking, and rapid re-acceleration sequences can stimulate neuromuscular adaptations relevant to multidirectional performance. Furthermore, while analytical training such as SAQ (Speed, Agility, and Quickness) may be superior for raw linear speed, SSGs are uniquely effective for enhancing perceptual-cognitive agility and decision-making under uncertainty [9, 20].

From a programming perspective, the evidence favors short work bouts (30–90 seconds) with brief recoveries (1:1 to 1:2 ratios) to maintain a high density of mechanical loading without the performance decay associated with metabolic fatigue [13, 17, 20, 38]. Rule-based constraints further amplify this stimulus; man-to-man marking, coach encouragement, and the use of multiple mini-goals (e.g., 2 or 4) increase movement urgency and force players to frequently reorient their movement to exploit defensive width [6, 16, 17, 19]. The “Score Zone” (stop-ball) rule is also highlighted as a potent lever for increasing mechanical intensity compared to traditional goals [6, 16].

From a physiological standpoint, the adaptations targeted by these compact formats reflect greater eccentric braking demands, increased stretch–shortening cycle loading, and rapid recruitment of high-threshold motor units [20]. Repeated exposure to these eccentric braking actions has also been associated with neuromuscular adaptations that improve deceleration control and change-of-direction efficiency. These mechanisms are optimized when players are exposed to repeated directional changes with incomplete recovery. For practitioners, the choice of format represents a decisive trade-off: while smaller formats maximize mechanical load and technical-tactical transitions, larger pitch sizes and formats necessarily shift the external load profile toward greater HSR and sprint exposure [8, 10, 17, 36]. This trade-off highlights the importance of combining different SSG formats within a training microcycle to target complementary physical qualities.

### Interpretation of Technical and Skill-Related Outcomes

This synthesis suggests that technical execution and skill acquisition can be preferentially targeted through small formats (1 v 1–4 v 4) combined with time–space–compressing task constraints [9, 42]. This pattern is consistent with the findings reported in the Results section, where smaller SSG formats produced the highest densities of individual technical actions. Reducing player numbers (e.g., from 4 v 4 to 2 v 2) has been shown to increase individual technical actions—such as passes, receptions, and shots—by approximately 60% [28, 43]. Specifically, the 3 v 3 format is uniquely effective for technical development, providing an optimal balance for successful shots, dribbling, and ball possession [27, 29]. Smaller relative player area and compact fields further promote individual ball engagement and more frequent technical-tactical transitions compared to larger, match-representative spaces [2, 19, 28].

Rule-based manipulations serve as high-impact design levers for steering the technical profile of a session. Touch limitations (e.g., 1- or 2-touch) significantly increase interaction density and the speed of ball circulation, forcing players to execute skills under higher time– space pressure [2, 6, 31]. However, practitioners should be mindful that strict touch limits—particularly 1-touch—often lead to higher technical error rates and reduced execution accuracy, especially in youth or less experienced populations [2, 28, 31]. Furthermore, directionality and scoring constraints selectively bias the practiced actions; 1-goal (1G) formats prioritize finishing and defensive coverage, whereas multiple mini-goals shift play toward lateral corridors, encouraging offensive variety and dribbling to exploit defensive width [16, 19].

From a programming perspective, maintaining quality and execution precision requires careful management of fatigue. The evidence suggests utilizing intermittent work bouts of 2.5 to 4 minutes [6, 23, 24, 29]. These durations, paired with adequate recovery (e.g., 1:1 ratios), help sustain a high volume of successful technical actions before performance decays due to high metabolic strain [3, 17]. Beyond physical load, the use of neutral players (jokers) can facilitate ball retention and increase passing volume, provided pitch dimensions are adjusted proportionally to maintain the desired technical density [2, 10, 19].

From a mechanistic and pedagogical standpoint, SSGs serve as primary skill-learning tasks because they foster perception–action coupling within representative environments [17, 24]. This principle aligns with the representative learning design framework, which emphasizes that skill acquisition is enhanced when training tasks preserve the informational constraints of competitive play. Observed technical improvements reflect context-specific repetition and enhanced attunement to relevant game cues. Implementation may be further supported by integrating differential learning, augmented feedback, and contextual interference strategies within the SSG constraints [3, 4]. Finally, practitioners must acknowledge the inherent trade-offs: while technically-dense micro-formats maximize skill repetitions, they simultaneously entail reduced high-speed running and sprint exposure, which must be addressed through the broader training plan [9, 17].

### Interpretation of Tactical Behaviour and Collective Organization

The present synthesis suggests that collective tactical behaviour in SSG is strongly influenced by the interaction between relative area per player, player format, and task constraints [8, 10, 17, 19]. This interpretation is consistent with the results of the present synthesis, which show that variations in pitch size and player configuration were associated with clear changes in team dispersion metrics. In line with the spatial–temporal outcomes, larger area per player and expanded formats are consistently associated with increases in team length, width, surface area, stretch index, and inter-player distances, indicative of more expansive team structures and pitch control [8, 10, 17, 19]. These responses indicate that spatial affordances scale with the available playing area, facilitating a wider exploration of functional solutions during possession and defensive organization [4, 10, 22]. Conversely, smaller formats (1 v 1–4 v 4) combined with restrictive constraints promote team compactness, shorter interaction cycles, and tighter local coupling between subunits [2, 4, 18, 19, 22].

This pattern aligns with constraint-led perspectives, in which reducing space or manipulating rules—such as touch limits and pressing triggers—narrows the solution space and biases behaviour toward coordinated actions at higher interaction frequencies [2, 6, 19, 31]. Within ecological dynamics frameworks, such constraint manipulations are understood to shape the affordances available to players, thereby guiding collective coordination without explicit prescriptive instruction. Evidence indicates that micro-formats (1 v 1 to 3 v 3) increase the density of individual tactical actions (e.g., penetration and defensive delays), accelerate decision-making, and promote exploratory behavior [1, 2, 5, 22]. Within this context, SSG designs with a smaller area per player serve as effective tools for reinforcing synchronized offensive–defensive transitions, particularly when tactical aims involve compactness or pressing traps [8, 9, 20].

Directionality and goalkeeper involvement emerge as critical secondary levers that modulate collective organization [16, 17, 19]. Directional play with a single goal (1G) has been shown to augment forward connectivity and bias spatial orientation along progression corridors, often resulting in more elongated structures and defensive grouping [16, 19, 22]. In contrast, multiple-goal configurations (e.g., 2 or 4 mini-goals) shift play toward lateral corridors to exploit defensive width, while possession-oriented rules produce more evenly distributed interaction networks [16, 17, 19]. Furthermore, increasing area per player and employing numerical imbalances (e.g., 3 v 4 or 4 v 5) elevate the variability and entropy of collective movements, fostering adaptable team behaviours through a wider repertoire of emergent tactical solutions [5, 12, 17, 21].

To preserve the accuracy of these collective behaviors, the evidence suggests maintaining work bouts between 2.5 and 4 minutes, as physical and mental fatigue specifically reduces the precision of offensive unity and synchronized defensive behaviors [3, 17, 20, 23, 26]. Overall, tactical behaviour is most effectively shaped through a combination of spatial scaling, structural design (format and directionality), and rule-based constraint manipulation [17, 19, 22]. Larger, directional designs tend to promote expansive structures and exploratory dynamics, whereas smaller, constraint-rich conditions favor compactness, coordinated pressure, and relatively stable interaction patterns [4, 17, 22].

### Practical Implications for Coaches

The integrated model and decision matrix presented in this review provide coaches with an evidence-informed framework to align SSG design with specific performance aims. Relative area per player emerges as the central tuning variable for regulating physical and physiological load. Specifically, larger areas (≥ 200 m^2^ · player^−1^) combined with directional formats and goalkeepers are essential for promoting high-speed running, while reaching maximal sprinting thresholds requires an area per player exceeding 300–365 m^2^. Conversely, smaller area per player values (60–120 m^2^ · player^−1^) combined with tighter task constraints bias training toward anaerobic bursts, higher ACC–DEC density, and increased technical repetitions.

Format selection should be guided by the intended tactical and physical scale. Micro-formats (1 v 1–3 v 3) are superior for maximizing individual ball engagement, explosive mechanical actions, and individual tactical skills such as penetration and duels. The 4 v 4 format is identified as a “gold standard” for balancing high cardiovascular strain with match-representative running volumes. For largerscale tactical principles, team compactness, and positional regularity, practitioners should utilize medium- to large-sided formats (6 v 6–10 v 10), which facilitate longer passing sequences and expansive collective organization.

Temporal constraints and work-to-rest (W:R) ratios represent additional precision levers. Aerobic power is best targeted through 3–6-minute work bouts (ideally 4-minute intervals) with 1:1 to 1:2 W:R ratios. In contrast, anaerobic and glycolytic demands require shorter, higher-intensity bouts of 20–45 seconds (up to 2 minutes) with similar recovery ratios to maintain metabolic pressure. When targeting high-speed running, coaches must shift to wider recovery windows (1:3 to 1:6 W:R ratios) to ensure players are sufficiently recovered to maintain peak velocities (≥ 90% Vmax) and avoid fatigue-driven suppression of sprint expression.

Rule-based modifiers, such as touch limitations (1- or 2-touch) and man-to-man marking, serve as precision tools to increase tempo, decision-making speed, and metabolic strain. Strategic joker usage can be employed to create numerical overloads and stabilize possession, provided pitch dimensions are adjusted proportionally to maintain movement urgency. Finally, because SSGs often provide inconsistent exposure to maximal linear speed, practitioners should selectively integrate complementary “top-up” conditioning—such as short HIIT intervals, repeated-sprint blocks, or isolated sprint drills— to bridge the gap between game-based loads and competitive match demands. Collectively, these principles enable coaches to construct goal-oriented, repeatable tasks that preserve ecological validity while maximizing training efficiency.

### Methodological Considerations, Limitations, and Future Research Directions

Several methodological considerations must be taken into account when interpreting the synthesized evidence and applying the proposed SSG design model. First, the certainty of evidence across existing systematic reviews and meta-analyses is constrained by heterogeneous study designs, inconsistent reporting of essential variables such as area per player, bout duration, work-to-rest structure, and scoring constraints, and frequent omission of contextual factors, including player level, tactical style, and microcycle periodization. Many interventions rely on small sample sizes, limited randomization, or short program durations, which may restrict the generalizability of findings across competitive levels. Positional-tracking outcomes, particularly tactical indices such as stretch index, surface area, or network centralities, are not reported consistently across studies, making cross-study comparisons difficult and limiting fine-grained inferences about collective organization. Additionally, wide prediction intervals for high-speed running and sprint exposure reflect substantial intra- and inter-study variability, challenging precise prescription and underscoring the need for context- and team-specific calibration.

Most studies tend to isolate single constraints rather than systematically assess multi-constraint interactions, limiting the ability to capture the emergent, nonlinear nature of tactical–physical coupling within SSG. Furthermore, considerable inter-individual variability in dose–response relationships has been documented, as players with differing fitness levels, tactical roles, and perceptual–cognitive capacities often respond differently to the same SSG format. This individual-level variability complicates generalization at the group level and underscores the importance of adaptive, player-specific monitoring strategies in both research and applied practice. Future research should prioritize the development and adoption of standardized reporting frameworks for SSG design, including explicit documentation of area per player, task constraints, spatial geometry, bout structure, and athlete characteristics. High-resolution positional tracking may be incorporated more consistently to better elucidate the links between constraint manipulation, collective organization, and performance outcomes. Multi-constraint experimental designs are needed to examine interaction effects and to more accurately reflect the ecological complexity of constraint-led approaches.

Longitudinal and periodized studies are warranted to examine how SSG-derived adaptations evolve across microcycles and competitive phases, and how complementary “top-up” methods (e.g., RSA, HIIT, or ACC-DEC-focused tasks) can be strategically integrated into broader training programs. Furthermore, research involving female athletes, youth cohorts, and elite professional populations remains limited and should be expanded to improve representativeness and external validity. Finally, future work may explore individualized or AI-assisted SSG prescription models that integrate player profiling, load monitoring, and tactical objectives to support decision-making in applied settings. Collectively, addressing these methodological gaps is expected to strengthen the empirical foundation of SSG research and to refine evidence-informed frameworks for performance-driven task design.

## CONCLUSIONS

This umbrella synthesis integrates evidence from 37 systematic reviews and meta-analyses to develop an evidence-informed framework linking core SSG design levers—relative area per player, format, bout duration, work-to-rest structure, task constraints, touch limitations, joker usage, and directionality/goalkeeper involvement—with predefined physical, technical, and tactical training aims. Across the reviewed literature, several patterns appear consistently across multiple syntheses and therefore represent the most robust conclusions of the present work. In particular, spatial density (area per player) and format size emerge as the primary structural regulators of physiological load and locomotor demands, while rule-based and informational constraints act mainly as secondary modifiers shaping technical actions and tactical behaviours.

Evidence consistently indicates that SSGs can elicit substantial aerobic loads and support endurance adaptations comparable to those of running-based high-intensity interval training when overall session exposure (e.g., total work duration and intensity) is closely matched between methods. High-speed running and sprint exposure are most strongly associated with larger relative playing areas and directional formats, whereas acceleration–deceleration and changeof-direction demands, together with high densities of technical actions, tend to be emphasized in smaller formats with reduced space and tighter task constraints. Tactical behaviours vary systematically with spatial and directional affordances, reflecting shifts in collective dispersion, coordination, and interaction patterns.

However, several conclusions should be interpreted with greater caution. Evidence on tactical metrics, elite professional cohorts, and female populations remains relatively sparse in the current literature, and overlap among systematic reviews may partially account for the apparent convergence of some findings. As a result, while the spatial and temporal determinants of physical load appear relatively robust, conclusions regarding complex tactical behaviours and certain contextual moderators should be considered more tentative and may require further empirical confirmation.

Importantly, the synthesis highlights that SSG task design inherently involves trade-offs between competing training objectives. Manipulating one constraint to enhance a specific outcome may simultaneously attenuate another (e.g., smaller formats increase technical engagement and mechanical load but reduce exposure to high-speed running). For this reason, SSG programming should be interpreted as a multi-objective optimization process, in which coaches balance spatial, temporal, and rule-based constraints according to the priorities of a given training session or microcycle, rather than applying a single fixed configuration.

The resulting decision matrix is intended as a structured, transparent design aid that organizes convergent evidence into a practitioner-oriented framework. Rather than representing fixed prescriptions, the matrix supports context-sensitive planning and encourages practitioners to align SSG design with specific performance objectives while considering player characteristics, monitoring data, and training periodization.

Finally, the proposed framework should be viewed as an updateable synthesis tool. As new systematic reviews, meta-analyses, and high-resolution tracking studies emerge—particularly in underrepresented populations such as elite female players and professional environments—the decision matrix can be refined and expanded. Continued empirical research examining interactions between SSG design variables will further strengthen the evidence base supporting performance-driven task design in soccer training.

## Supplementary Material

An umbrella review of soccer small-sided games: developing an evidence-informed design model linking training components to performance outcomes

An umbrella review of soccer small-sided games: developing an evidence-informed design model linking training components to performance outcomes

## Data Availability

Materials are available on request from the corresponding author.

## Supplementary Materials

Link to Table S1, Link to Table S2.
