# Supplementary material for: An umbrella review of soccer small-sided games: developing an evidence-informed design model linking training components to performance outcomes
Source: Biol Sport. 2026 Jun 10;43:1487–522. doi: 10.5114/biolsport.2026.161109 (PMC13617480; doi:10.5114/biolsport.2026.161109)
Supplement: Supplementary file 1 [file JBS-43-57927-s1.pdf]

Supplementary Material

To article: Ulupınar S, Özbay S, Ateş O et al. An umbrella review of soccer small-sided games: developing an evidence-informed design model linking training components to performance outcomes. Biol Sport. 2026;43:1487–1522. DOI: https://doi.org/10.5114/biolsport.2026.161109

Database-Specific Search Strategies

A comprehensive literature search was conducted to identify systematic reviews and meta-analyses examining the effects of sided games and related game-based training formats in soccer. Searches were performed in Web of Science, PubMed, Scopus, and SPORTDiscus. The search strategies were designed to capture the wide range of terminology used for small-sided games and related formats in soccer training. The final search queries used in each database are presented below.

Web of Science

TS=(("small-sided gam*" OR "sided gam*" OR SSG OR "modified gam*" OR "conditioned gam*"

OR "game-based training" OR "sided format*" OR "area per player" OR ApP OR "pitch size")

AND (soccer OR football)

AND ("systematic review" OR "meta-analysis" OR "meta analysis"

OR "umbrella review" OR "quantitative review"

OR "meta-regression" OR "evidence synthesis"))

PubMed

(("small-sided gam*"[Title/Abstract] OR "sided gam*"[Title/Abstract]

OR SSG[Title/Abstract] OR "modified gam*"[Title/Abstract]

OR "conditioned gam*"[Title/Abstract] OR "game-based training"[Title/Abstract]

OR "sided format*"[Title/Abstract] OR "area per player"[Title/Abstract]

OR ApP[Title/Abstract] OR "pitch size"[Title/Abstract])

AND (soccer[Title/Abstract] OR football[Title/Abstract]))

AND ("systematic review"[Publication Type] OR "meta-analysis"[Publication Type]

OR "systematic review"[Title/Abstract] OR "meta-analysis"[Title/Abstract])

Scopus

TITLE-ABS-KEY(("small-sided gam*" OR "sided gam*" OR SSG

OR "modified gam*" OR "conditioned gam*" OR "game-based training"

OR "sided format*" OR "area per player" OR ApP OR "pitch size")

AND (soccer OR football)

AND ("systematic review" OR "meta-analysis"

OR "umbrella review" OR "quantitative review"

OR "meta-regression"))

SPORTDiscus

(("small-sided gam*" OR "sided gam*" OR SSG OR "modified gam*"

OR "conditioned gam*" OR "game-based training" OR "sided format*"

OR "area per player" OR ApP OR "pitch size")

AND (soccer OR football)

AND ("systematic review" OR "meta-analysis"

OR "umbrella review" OR "quantitative review"))

Additional Sources

To ensure comprehensive coverage, additional studies were identified through Google Scholar searches and manual screening of reference lists from relevant systematic reviews and meta-analyses.
